# Key roles for phosphorylation and the Coiled-coil domain in TRIM56-mediated positive regulation of TLR3-TRIF–dependent innate immunity

**DOI:** 10.1016/j.jbc.2024.107249

**Published:** 2024-03-29

**Authors:** Benjamin M. Liu, Nan L. Li, Ruixue Wang, Xiaofan Li, Z. Alex Li, Tony N. Marion, Kui Li

**Affiliations:** 1 Department of Microbiology, Immunology and Biochemistry, University of Tennessee Health Science Center, Memphis, Tennessee, USA; 2 Divisions of Pathology and Laboratory Medicine, Children’s National Hospital, Washington, District of Columbia, USA; 3 Department of Pediatrics, The George Washington University School of Medicine and Health Sciences, Washington, District of Columbia, USA; 4 Department of Pathology, The George Washington University School of Medicine and Health Sciences, Washington, District of Columbia, USA; 5 Department of Microbiology, Immunology and Tropical Medicine, The George Washington University School of Medicine and Health Sciences, Washington, District of Columbia, USA; 6 Children’s National Research Institute, Washington, District of Columbia, USA; 7 The District of Columbia Center for AIDS Research, Washington, District of Columbia, USA

**Keywords:** TRIM56, Toll-like receptor-3, TRIF, IRF3, NF-kappaB, phosphorylation, interferon, Coiled-coil domain, antiviral state

## Abstract

Tripartite-motif protein-56 (TRIM56) positively regulates the induction of type I interferon response *via* the TLR3 pathway by enhancing IRF3 activation and depends on its C-terminal residues 621-750 for interacting with the adaptor TRIF. However, the precise underlying mechanism and detailed TRIM56 determinants remain unclear. Herein, we show ectopic expression of murine TRIM56 also enhances TLR3-dependent interferon-β promoter activation, suggesting functional conservation. We found that endogenous TRIM56 and TRIF formed a complex early (0.5–2 h) after poly-I:C stimulation and that TRIM56 overexpression also promoted activation of NF-κB by poly-I:C but not that by TNF-α or IL-1β, consistent with a specific effect on TRIF prior to the bifurcation of NF-κB and IRF3. Using transient transfection and Tet-regulated cell lines expressing various TRIM56 mutants, we demonstrated the Coiled-coil domain and a segment spanning residues ∼434-610, but not the B-box or residues 355-433, were required for TRIM56 augmentation of TLR3 signaling. Moreover, alanine substitution at each putative phosphorylation site, Ser^471^, Ser^475^, and Ser^710^, abrogated TRIM56 function. Concordantly, mutants bearing Ser^471^Ala, Ser^475^Ala, or Ser^710^Ala, or lacking the Coiled-coil domain, all lost the capacity to enhance poly-I:C–induced establishment of an antiviral state. Furthermore, the Ser^710^Ala mutation disrupted the TRIM56-TRIF association. Using phospho-specific antibodies, we detected biphasic phosphorylation of TRIM56 at Ser^471^ and Ser^475^ following TLR3 stimulation, with the early phase occurring at ∼0.5 to 1 h, prior to IRF3 phosphorylation. Together, these data reveal novel molecular details critical for the TRIM56 augmentation of TLR3-dependent antiviral response and highlight important roles for TRIM56 scaffolding and phosphorylation.

In response to viral insults, the innate immune system constitutes a front line of host defense armed with multi-layered mechanisms (1, 2, 3, 4, 5, 6, 7, 8, 9, 10). Of these, pattern recognition receptors, such as Toll-like receptors (TLRs) and retinoic-inducible gene-I–like receptors, play important roles in recognizing viral nucleic acids as a major class of viral pathogen-associated molecular pattern and triggering intracellular signaling pathways that culminate in activation of interferon regulatory factor 3 (IRF3) and NF-κB (1, 2, 3, 4, 5, 6, 7, 8). These two transcription factors play pivotal parts in the production of interferons (IFNs) and pro-inflammatory cytokines/chemokines, respectively (1, 2, 3, 4, 5, 6, 7). Type I (IFN-β and IFN-α) and predominantly on mucosal surfaces, type III (IFN-λs, a.k.a, IL29, IL28A, and IL28B, etc) IFNs further induce hundreds of IFN-stimulated genes (ISGs) that collectively establish an antiviral state, restricting viral propagation and spread (1, 2, 3, 4, 5, 6, 7).

Over the past 2 decades, members of the tripartite motif protein (TRIM) family as intracellular restriction factors are increasingly recognized as active players in antiviral innate immunity (11, 12, 13, 14, 15, 16, 17, 18). Structurally, TRIMs possess highly conserved arrangement of domains in the N-terminal RBCC motif (*i.e.*, RING, B-box, and Coiled-coil domains), but their C-terminal portions vary (11, 12, 13). In general, RING domains confer TRIMs with E3 ubiquitin ligase activity. B-box domains are considered zinc-binding motifs while Coiled-coil domains facilitate self-association and oligomerization to act as scaffolds for recruitment and formation of multi-protein complexes (11, 12, 13). Much attention has been paid to delineating direct antiviral roles of the TRIMs in contributing to host restriction of some specific types of viruses by targeting specific steps in the viral life cycle. Our previous work has revealed that TRIM56 is a pleiotropic host antiviral factor restricting distinct RNA viruses including members of the family *Flaviviridae* (bovine viral diarrhea virus, yellow fever virus, dengue virus, and Zika virus) (15, 17, 19), influenza A and B virus (18), and human coronavirus-OC43 (hCoV-OC43) (17), *via* overlapping and distinct molecular determinants and by targeting viral RNA replication or a later step, although the exact TRIM56 targets responsible for the observed antiviral effects have not been identified.

Recently, “indirect” antiviral roles of subsets of TRIMs, specifically in regulating innate antiviral immune signaling pathways—through general augmentation of the IFN response—have begun to be appreciated (11, 12). Approximately, half of the ∼75 TRIMs can act at different levels in innate immune signaling, thereby regulating antiviral responses (11, 14, 16, 20, 21, 22). Our lab has demonstrated that TRIM56 promotes TLR3-dependent IRF3 activation *via* a non-canonical mechanism that is independent of its E3 ligase activity and RING domain but, instead, requires its C-terminal integrity that we found to be important for association with the TLR3 adaptor protein, that is, TIR-domain-containing adapter-inducing IFN-β (TRIF) (14). However, the precise underlying mechanism and detailed determinants governing this function of TRIM56 remains unclear. In this study, we set out to investigate whether the ability to positively regulate TLR3 signaling is conserved between human and murine TRIM56 and to map the domains and residues in TRIM56 that are critically required, in efforts to understand the underpinning biology. Our data highlight the important roles for the Coiled-coil domain and specific phosphorylations and reveal novel molecular details of TRIM56-mediated upregulation of TLR3-dependent antiviral immunity.

## Results

### Murine TRIM56 promotes activation of the IFN-β promoter downstream of the TLR3 pathway

**Figure 1:**
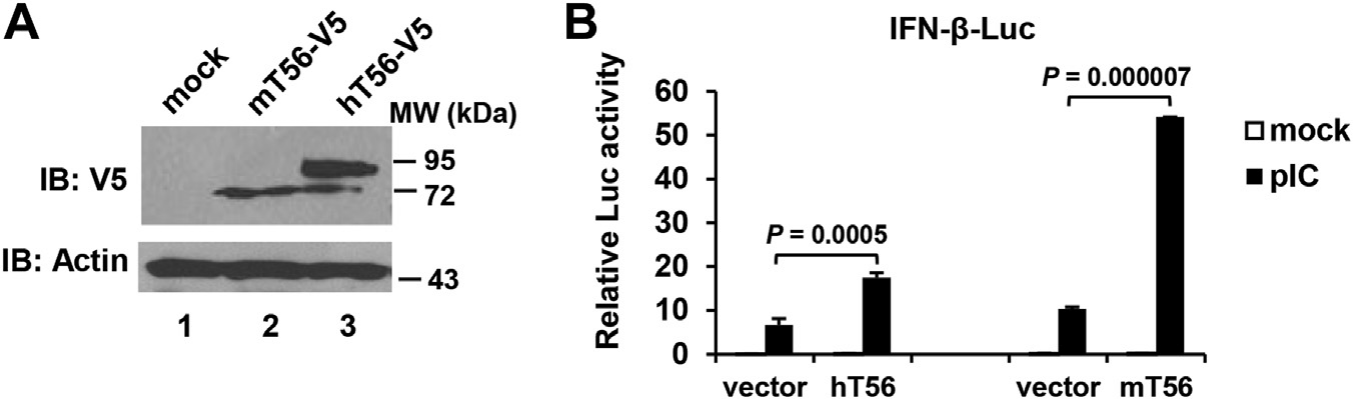
**Murine TRIM56 augments activation of the IFN-β promoter *via* the TLR3 pathway.***A*, immunoblot analysis of the expression of vectors encoding C-terminally V5-tagged murine TRIM56 (mT56-V5) and human TRIM56 (hT56-V5), respectively, in transfected HEK293 cells using mouse anti-V5 mAb. Actin serves as the loading control. *B*, HEK293-TLR3 cells were cotransfected with a plasmid encoding hT56 or mT56 or the corresponding empty vector (vector), as well as internal control plasmid pRL-TK and reporter plasmid IFN-β-Luc, followed by mock stimulation or stimulation by poly-I:C (pIC) for 8 h. Dual luciferase reporter assay was then performed to measure the promoter activation. *p* values for the comparison between vector and hT56 or mT56 group were indicated. IFN, interferon; TLR, Toll-like receptor; TRIM56, tripartite-motif protein-56.

We have previously demonstrated that human TRIM56 (here referred to as hTRIM56, hT56, TRIM56, or T56) is a positive regulator of IRF3-dependent antiviral response *via* the TLR3 pathway (14). Sequence alignment revealed that there is 81% amino acid (aa) homology between hTRIM56 and murine TRIM56 (here referred to as mTRIM56 or mT56), although the latter is 734-aa-long, short of 21 aa compared to the former (Fig. S1). Notably, residues 411-416 and 422-437 in hTRIM56 are absent in mTRIM56; Pro^373^ in mTRIM56, on the other hand, is absent in its human counterpart (Fig. S1). We set out to investigate if mTRIM56 has similar capacity to hTRIM56 in modulating TLR3 signaling. To this end, the impact of ectopic expression of mTRIM56 or hTRIM56 on extracellular poly-I:C–induced IFN-β promoter activation was determined. Immunoblotting data (Fig. 1*A*) showed that mTRIM56 protein migrated a little faster than hTRIM56 on SDS-PAGE, consistent with it being a slightly shorter protein. Compared with the empty vector control, both mTRIM56 and hTRIM56 significantly enhanced activation of the IFN-β promoter following poly-I:C stimulation (Fig. 1*B*). Interestingly, a more potent effect of mTRIM56 was observed. Specifically, we found mTRIM56 augmented TLR3 signaling by ∼5-fold (*p* < 0.00001), while the enhancement by hTRIM56 was ∼2.6-fold under the same experimental conditions (*p* < 0.001)—consistent with our previous report (14). Thus, the ability of TRIM56 to augment TLR3-dependent type I IFN activation is conserved between human and mouse.

### TRIM56 promotes activation of both IRF3 and NF-κB branches downstream of TLR3 signaling by forming a complex with TRIF early after engagement of the pathway

**Figure 2:**
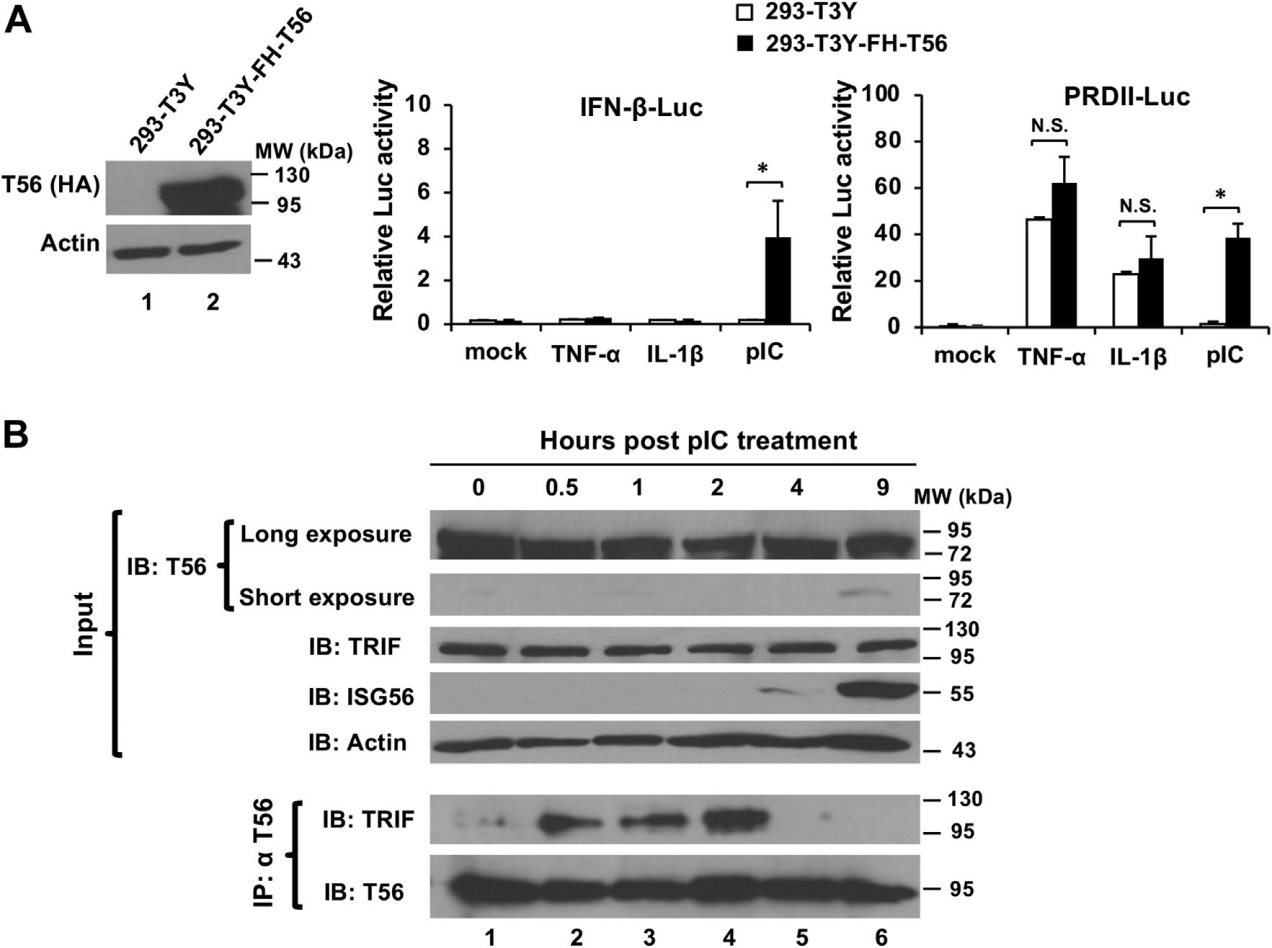
**TRIM56 potentiates activation of both IRF3 and NF-κB branches downstream of TLR3 signaling by forming a complex with TRIF early after engagement of the pathway.***A*, (*Left* panel) immunoblot analysis of the expression of N-terminally Flag-HA-tagged TRIM56 (FH-T56) using mouse anti-HA mAb in HEK293-TLR3-YFP (293-T3Y)-derived stable cells (293-T3Y-FH-T56) overexpressing FH-T56. HEK293-T3Y-FH-T56 and its parental 293-T3Y cells were cotransfected with internal control plasmid pRL-TK and reporter plasmid IFN-β-Luc (*middle* panel) or NF-κB responsive reporter plasmid PRDII-Luc (*right* panel), followed by mock treatment or treatment with TNF-α, IL-1β, or poly-I:C for 8 h. Dual luciferase reporter assay was then performed to measure the corresponding promoter activation. *B*, co-immunoprecipitation analysis of the interaction of endogenous TRIM56 with endogenous TRIF in HeLa cells at indicated time points post poly-I:C stimulation. Cell lysates were immunoprecipitated (IP) with anti-TRIM56 pAb, followed by immunoblotting (IB) with anti-TRIF or anti-TRIM56. The *upper* panels show expression of TRIM56, TRIF, IFN-stimulated gene 56 (ISG56), and actin in cell lysates. Single asterisk indicates that statistical differences exist between mock- and poly-I:C-treated cells with a *p* value of < 0.05. N.S., not statistically significant; pIC, poly-I:C. IFN, interferon; IRF, interferon regulatory factor; TLR, Toll-like receptor; TRIM56, tripartite-motif protein-56.

In a previous study (14), we reported that TRIM56 and TRIF formed a complex when ectopically co-expressed in cells. It was proposed this capacity of TRIM56 underlies heightened IRF3 activation and ISG induction *via* the TLR3 pathway. Since engagement of TLR3 leads to activation of IRF3 as well as NF-κB and TLR3 signaling bifurcates at the adaptor protein TRIF, we reasoned that the TRIM56–TRIF interaction may also impact the NF-κB branch downstream this pathway. To test this, we compared human embryo kidney (HEK) 293 cells stably expressing low levels of TLR3-YFP (293-T3Y) and their derived cells with stable, ectopic expression of FLAG- and HA-tandem tagged TRIM56 (293-T3Y-FH-T56) (Fig. 2*A*, left) for responses to TNF-α, IL-1β, and extracellular poly-I:C, respectively. The results confirmed the ability of TRIM56 to augment activation of the IFN-β promoter by poly-I:C (Fig. 2*A*, middle), consistent with our previous report (14). When we examined activation of the NF-κB–dependent PRDII promoter, we found that poly-I:C was significantly more effective in 293-T3Y-FH-T56 cells than in control 293-T3Y cells (Fig. 2*A*, right, *p* < 0.05). Specifically, compared to the mock-treatment group, poly-I:C stimulation led to ∼4-fold increase in PRDII promoter activity in control 293-T3Y cells. Strikingly, the TLR3 ligand stimulated PRDII promoter by ∼75-fold in 293-T3Y-FH-T56 cells, compared with cells under mock-treated condition. In contrast, when compared to mock-stimulated groups, TNF-α activated the PRDII promoter by ∼132-fold and ∼122-fold in control 293-T3Y and 293-T3Y-FH-T56, respectively, whereas IL-1β upregulated the promoter activity by ∼66-fold in control 293-T3Y and by ∼58-fold in 293-T3Y-FH-T56 (Fig. 2*A*, right). Therefore, PRDII promoter was activated to similar extent following stimulation by TNF-α or IL-1β, regardless of TRIM56 overexpression, suggesting that TRIM56 promotes NF-κB activation *via* the TLR3-TRIF pathway but has no effect on NF-κB–activating pathways that are TRIF-independent. These data are also in line with our previous proposed model in which TRIM56 acts through TRIF (14).

To understand the dynamics of the TRIM56–TRIF interaction under physiological conditions during TLR3 signaling, we performed co-immunoprecipitation (co-IP) analysis of endogenous TRIM56 and TRIF proteins in HeLa cells at different times post stimulation by poly-I:C. We observed early, transient association of TRIM56 and TRIF between 0.5 h and 2 h, which returned to background levels thereafter (Fig. 2*B*). It is worth noting that upregulation of ISG56, a well-characterized IRF3 target and sensitive readout for activation of the latent transcription factor, did not occur until 4 h poststimulation and later. Taken together, data from the aforementioned experiments demonstrate that the TRIM56–TRIF complex formation is an early event following TLR3 engagement and leads to heighted activation of both IRF3 and NF-κB signaling branches downstream of this pathway.

### TRIM56’s Coiled-coil domain and the C-terminal residues 434-610, but not the B-box or residues 355-433, are required for augmenting TLR3 signaling

**Figure 3:**
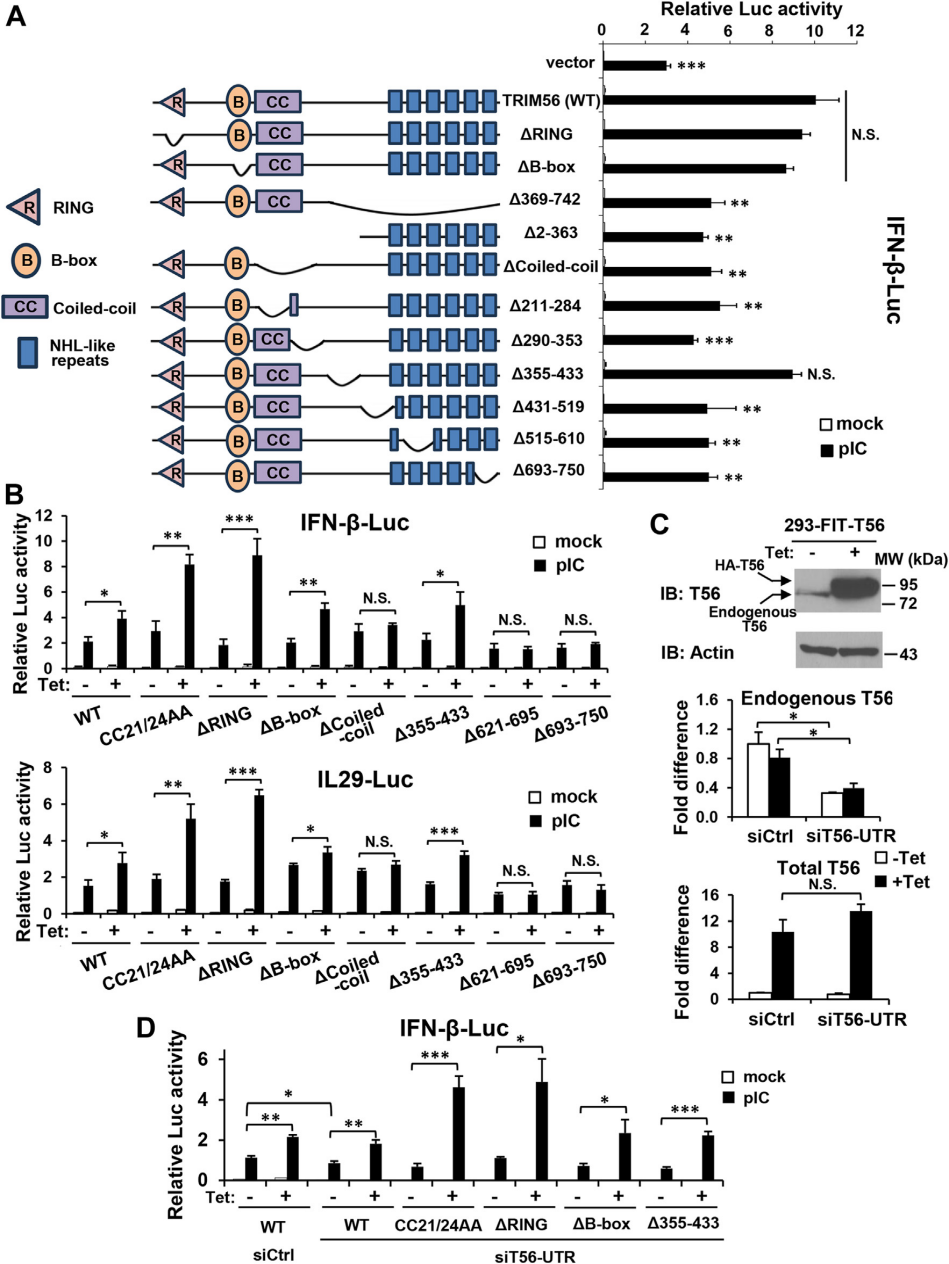
**Domain mapping of TRIM56 determinants required for its augmentation of TLR3 signaling to activation of IFN promoters.***A*, (*Left* panel) schematic representation of WT TRIM56 and various deletion mutants. R, B, and CC denote RING, B-box, and Coiled-coil domains, respectively. C-terminal putative NHL-like repeats are depicted as filled *blue* boxes. (*Right* panel) HEK293-TLR3 cells were cotransfected with an empty vector or a plasmid encoding WT TRIM56 or the indicated mutant, along with internal control plasmid pRL-TK and reporter plasmid IFN-β-Luc, followed by mock- or poly-I:C-stimulation for 8 h and dual luciferase reporter assay. *B*, Tetracycline (Tet)-inducible HEK293-Flp-In-T Rex (FIT)-derived cells conditionally expressing HA-tagged, WT, or indicated mutant (mut) TRIM56 (HEK293-FIT-T56 WT/Mut) cultured in the absence (−Tet) or presence (+Tet) of Tet were co-transfected with TLR3-encoding vector, pRL-TK and reporter plasmid IFN-β-Luc (*upper* panel) or IL-29-Luc (*lower* panel), followed by mock- or poly-I:C-stimulation for 8 h and dual luciferase reporter assay. *C*, (*Top* panel) immunoblot analysis of the expression of endogenous TRIM56 and exogenously overexpressed HA-TRIM56 (using anti-TRIM56) in HEK293-FIT-T56 cells with (+Tet) or without (−Tet) Tet addition for 48 h. qRT-PCR was carried out to measure the abundance of endogenous TRIM56 transcript (*middle* panel, using primers amplifying the 3′ UTR of TRIM56 mRNA), total TRIM56 transcript including the endogenous and exogenously overexpressed TRIM56 mRNAs (*bottom* panel, using primers amplifying the coding region of TRIM56 mRNA) in 293-FIT-T56 cells cultured with (+Tet) or without (−Tet) Tet, and transfected with a nontargeting control siRNA (siCtrl) or siRNA targeting 3′ UTR of TRIM56 (siT56-UTR). Of note, siT56-UTR silenced the expression of endogenous TRIM56 but not that of the exogenously introduced (Tet-inducible) TRIM56, which expressed only the coding region of the gene. *D*, HEK293-FIT-T56 WT/Mut cells cultured with (+Tet) or without (−Tet) Tet were transfected with siCtrl or siT56-UTR siRNA, along with a TLR3-encoding vector, pRL-TK and IFN-β-Luc, followed by mock- and poly-I:C-stimulation for 8 h and dual luciferase reporter assay. Statistical analysis was performed between WT TRIM56 and vector or mutant TRIM56 after poly-I:C stimulation in (*A*), between poly-I:C-treated -Tet and +Tet cells in (*B*) or (*D*), or between siCtrl and siT56-UTR groups in (*C*) or (*D*). Single, double, and triple asterisks denote that statistical differences exist with a *p* value of < 0.05, < 0.01, and < 0.001, respectively. N.S., not statistically significant; pIC, poly-I:C. IFN, interferon; TLR, Toll-like receptor; TRIM56, tripartite-motif protein-56.

We have previously demonstrated that a region close to its C-terminal end, that is, aa 621-750, but not the RING domain or E3 ligase activity, is critical for TRIM56-mediated positive regulation of IRF3-dependent signaling downstream of TLR3 (14). However, the detailed molecular determinants governing this biological property remain to be fully elucidated. To map the TRIM56 regions/motifs required for enhancing TLR3 signaling, we measured IFN-β promoter activation in cells transiently transfected with WT or various mutant forms of V5-tagged hTRIM56 (designated TRIM56 or T56), in comparison with an empty control vector, followed by with or without stimulation by extracellular dsRNA (Fig. 3*A*). The mutants investigated in these experiments included those lacking the RING (referred to as ΔRING), B-box (referred to as ΔB-box), or the entire (ΔCoiled-coil) or a part (Δ211–284 and Δ290–353) of the Coiled-coil domain, respectively, and mutants with different deletions in the C-terminal portion, including Δ355-433 (lacking aa 355–433), Δ431-519 (lacking aa 431–519), Δ515-610 (lacking aa 515–610), Δ693-750 (lacking aa 693–750), and Δ369-742 (lacking aa 369–742). We also included Δ2-363 (lacking aa 2–363), which is devoid of the N-terminal RBCC motif and part of the C-terminal portion. Successful expression of these V5-tagged TRIM56 mutants have been confirmed by immunoblotting and found to be comparable to that of WT TRIM56 (Fig. S2) (15). In agreement with our previous publication (14), the ΔRING mutant retained the ability to promote TLR3 signaling, while the mutants with deletions of (close to) the C-terminal end region (Δ693–750 and Δ369–742) were abrogated for the function (*p* < 0.01, Fig. 3*A*). Interestingly, we found the TRIM56’s C-terminal portion required for positive regulation of TLR3 signaling extended upstream to include also aa 434-610, since Δ431-519 and Δ515-610, but not Δ355-433, failed to augment TLR3-mediated IFN-β promoter activation, compared with WT TRIM56 (*p* < 0.01, respectively). As far as the N-terminal region is concerned, ΔB-box retained the ability to enhance IFN-β promoter activity after TLR3 engagement, while Δ2-363 lacking the entire RBCC motif did not. This latter observation thus suggested a role for the Coiled-coil domain. Indeed, deletion of the entire (ΔCoiled-coil) or either the N- (Δ211–284) or C-terminal (Δ290–353) portion of the Coiled-coil domain abolished the TRIM56 function (*p* < 0.01 to < 0.001). In aggregate, these data establish that not only a large chunk of the C-terminal portion (starting ∼aa 434, or about two fifths) of TRIM56 is essential but also the entire Coiled-coil domain is required for boosting TLR3 signaling. On the other hand, the B-box domain is dispensable, as is a central portion spanning aa 355-433, which is the least conserved region between human and murine TRIM56 (Fig. S1).

To corroborate these findings, we employed HEK293-Flp-In T-REx (FIT)-derived stable cell lines with tetracycline (Tet)-inducible expression of HA-tagged, WT TRIM56 or individual TRIM56 mutants of interest (Fig. 3*B*) and determined how conditional overexpression of each TRIM56 mutants would impact the activation of IFN-β and IL-29 (a.k.a., IFN-λ1) promoters *via* the TLR3 pathway (Fig. 3*B*). The expression levels of the HA-tagged TRIM56 mutants had been confirmed to be comparable to that of WT HA-TRIM56 in the induced state (*i.e.*, after Tet addition to culture medium, data not shown; (14, 17)). Because the HEK293-FIT cells were poor responders to extracellular poly-I:C stimulation due to very low expression of TLR3 (17), cells were co-transfected with a TLR3-encoding vector for all conditions in the IFN promoters activation experiments. In agreement with our previous findings (14), cells with Tet-induction (+Tet) of WT TRIM56 expression were significantly enhanced for poly-I:C–stimulated promoter activities for both IFN-β (Fig. 3*B*, upper panel) and IL-29 (lower panel), compared with cells cultured without Tet (−Tet), while Δ693-750 or Δ621-695 nullified TRIM56’s function. Interestingly, ΔRING and the E3 ligase-dead CC21/24AA mutants exhibited greater capacity to promote TLR3 signaling than WT TRIM56 (Fig. 3*B*). Consistent with the data from transient expression experiments (Fig. 3*A*), Tet-induced expression of ΔB-box and Δ355-433 significantly heightened TLR3 responses, while induction of ΔCoiled-coil failed to do so for either promoter reporter (Fig. 3*B*), resembling the effects of the C-terminal deletion mutants Δ693-750 and Δ621-695. These data substantiate the importance of the Coiled-coil domain to TRIM56-mediated positive modulation of the IFN response downstream of the TLR3 pathway.

TRIM56 can oligomerize and Coiled-coil domains have been shown to mediate the formation of anti-parallel dimmers for TRIM molecules (15, 23). To scrutinize the intact abilities of ΔB-box and Δ355-433 mutants to modulate TLR3 signaling, we need to exclude the possibility that these mutants retain their function by pairing with endogenous WT TRIM56. Our previous (17) and current qPCR data (Fig. 3*C*) showed the mRNA level of the exogenously introduced HA-tagged TRIM56 in cells cultured in the presence of Tet (+Tet) was ∼10-15-fold higher than that of the endogenous TRIM56 transcript (in −Tet cells). Accordingly, as shown in Figure 3*C* (top), the protein data were in line with the mRNA data—in −Tet cells, only endogenous TRIM56 was detected, while in +Tet cells, both forms of TRIM56 were visible. Densitometry analysis of the immunoblotting data show that the total TRIM56/actin arbitrary units for +Tet cells were ∼12-fold of that for −Tet cells. To efficiently deplete endogenous WT TRIM56, an siRNA (siT56-UTR) specifically targeting the 3′ UTR of the TRIM56 transcript was utilized. Compared to a non-targeting control siRNA (siCtrl), siT56-UTR significantly knocked down the expression of endogenous TRIM56 (by ∼70%, *p* < 0.05) in 293-FIT-T56 cells regardless of Tet addition (Fig. 3*C*, middle) but not to affect total TRIM56 abundance in +Tet cells, vast majority of which was exogenously expressed HA-TRIM56 having no 3′UTR sequence attached (Fig. 3*C*, bottom). We observed siT56-UTR significantly decreased poly-I:C–stimulated IFN-β promoter activity in 293-FIT-T56 cells cultured without Tet, when compared with the negative control siRNA (Fig. 3*D*; compare −Tet TRIM56-WT cells between siCtrl and siT56-UTR, filled bars). However, siT56-UTR-transfected 293-FIT-T56 cells cultured in the presence of Tet (+Tet) still displayed significantly (*p* < 0.01) higher IFN-β promoter activation than −Tet cells in response to poly-I:C stimulation, suggesting that endogenous TRIM56 depletion did not affect the function of the Tet-induced, exogenous HA-TRIM56 in promoting TLR3 signaling. This was also the case in 293-FIT-T56-CC21/24AA, -ΔRING, -ΔB-box, and Δ355-433 cell lines with Tet-regulated expression of the respective mutant. Similar observations were made when we examined the effects on activation of the IL-29 promoter in lieu of IFN-β (data not shown). These results imply that these functional TRIM56 mutants do not depend on pairing with the endogenous WT counterpart. In addition, they confirm that TRIM56’s B-box and aa 355-433 are dispensable for positive regulation of TLR3 signaling to IFN activation.

**Figure 4:**
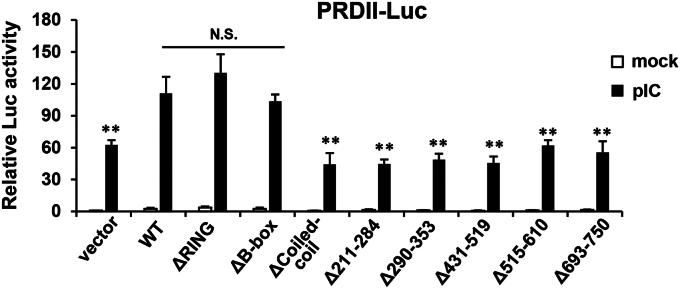
**Domain mapping of TRIM56 determinants required for its augmentation of TLR3 signaling to activation of NF-κB–dependent promoter.** HEK293-TLR3 cells were transfected with an empty vector (“vector”) or the indicated plasmid encoding WT or various mutant TRIM56-V5, along with internal control plasmid pRL-TK and NF-κB responsive reporter plasmid PRDII-Luc, followed by mock- or poly-I:C-stimulation for 8 h and dual luciferase reporter assay. Statistical analysis was performed between WT TRIM56 and vector or mutant TRIM56 after poly-I:C stimulation. Double asterisks denote that statistical differences exist with a *p* value of < 0.01. N.S., not statistically significant; pIC, poly-I:C. TLR, Toll-like receptor; TRIM56, tripartite-motif protein-56.

Importantly, our reporter gene assay experiments revealed the same domain requirement profile for the effect of TRIM56 on TLR3-dependent NF-κB activation (Fig. 4)—the integrity of the Coiled-coil domain and the C-terminal portion starting ∼residue 434 were both found to be required for heightening the activation of NF-κB–dependent PRDII promoter, while the RING or B-box was dispensable. These observations again reinforce the notion that TRIM56 acts on the TLR3 pathway prior to the bifurcation of NF-κB and IRF3.

### Putative phosphorylation sites Ser^471^, Ser^475^, and Ser^710^ in the C-terminal portion of hTRIM56 are critical for positive regulation of TLR3 signaling

**Figure 5:**
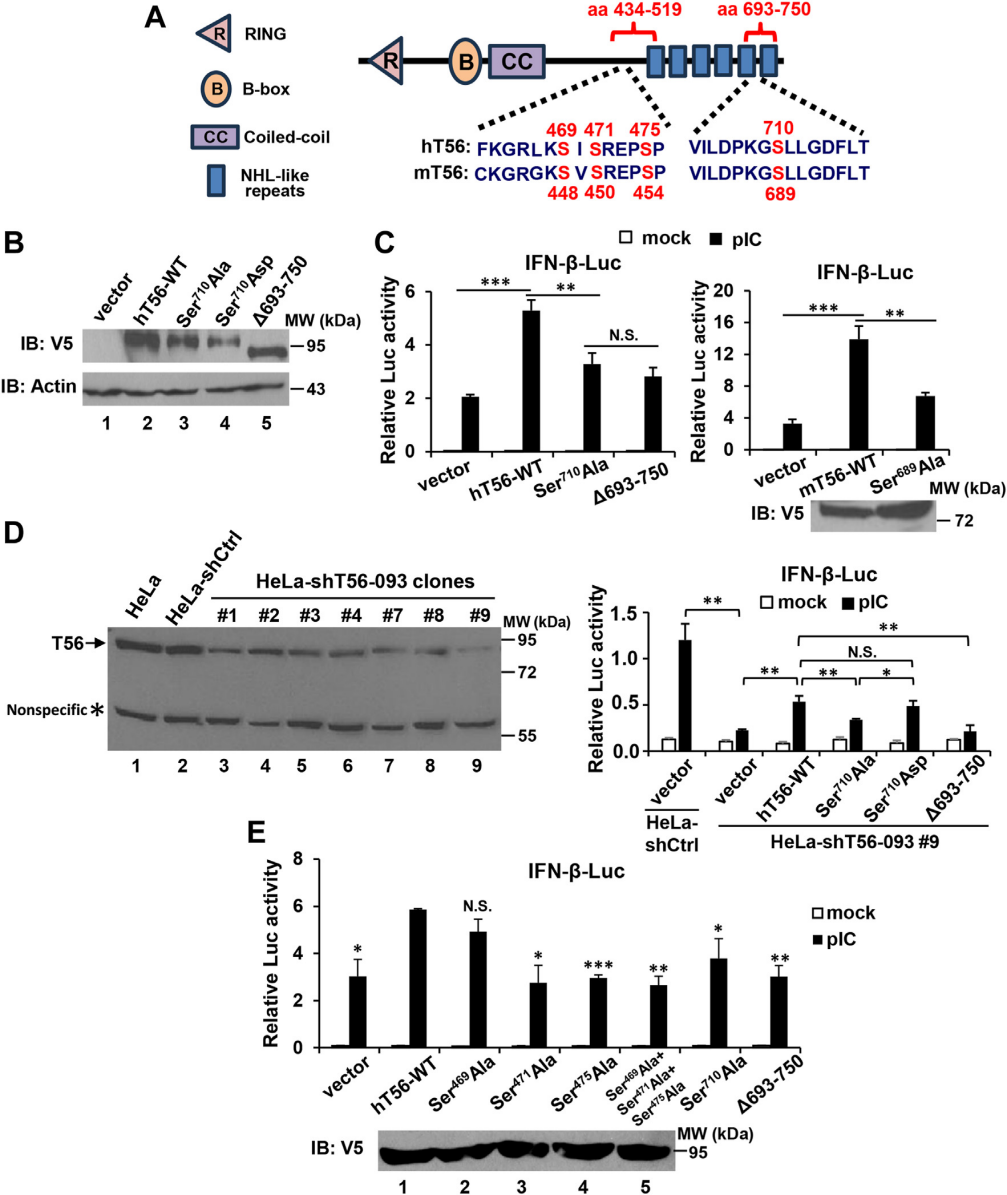
**Impact of mutations of putative phosphorylation sites Ser**^**471**^**, Ser**^**475**^**, and Ser**^**710**^**in the C-terminal portion of TRIM56 on the activation of IFN-β promoter downstream of TLR3 signaling.***A*, schematic representation of four conserved putative phosphorylation sites in human TRIM56 (hT56) and murine TRIM56 (mT56). The cluster of Ser^469^, Ser^471^, and Ser^475^ (Ser^448^, Ser^450^, and Ser^454^ in mT56) and Ser^710^ (Ser^689^ in mT56) fall into aa 434-519 and aa 693-750, respectively, regions critical for TRIM56 to potentiate TLR3 signaling (Figs. 3*A* and 5, (14)). *B*, immunoblot analysis of the expression of vectors encoding WT TRIM56-V5 and its Ser^710^Ala, Ser^710^Asp, and Δ693-750 mutants (using mouse anti-V5 mAb) in HEK293 cells. Actin serves as a loading control. *C*, HEK293-TLR3 cells were transfected with an empty vector (“vector”) or plasmids encoding WT or the indicated mutant hT56 (*left* panel) or WT or indicated mutant mT56 (*upper right* panel), along with internal control plasmid pRL-TK and reporter plasmid IFN-β-Luc, followed by mock stimulation or stimulation by poly-I:C for 8 h. Dual luciferase reporter assay was then performed to measure the promoter activation. (*Lower right* panel) Immunoblot analysis of the expression of vectors encoding WT mT56-V5 and its Ser^689^Ala mutant. *D*, (*Left* panel) immunoblot analysis of endogenous TRIM56 (using rabbit anti-TRIM56 pAb) in HeLa cells or HeLa stably transduced with a nontargeting, scrambled control shRNA (HeLa-shCtrl) or HeLa-shT56 cell clones stably transduced with a TRIM56 shRNA (HeLa-shT56-093) that targets the 3′UTR of human TRIM56 mRNA. Asterisk denotes a nonspecific band, which serves as a loading control. (*Right* panel) HeLa-shT56-093 #9 (*i.e.*, clone #9) and HeLa-shCtrl cells were transfected with an empty vector (“vector”) or a plasmid vector encoding WT or the indicated mutant human TRIM56, along with internal control plasmid pRL-TK and reporter plasmid IFN-β-Luc, followed by mock stimulation or stimulation by poly-I:C for 8 h. Dual luciferase reporter assay was then performed. *E*, (*Upper* part) human hepatoma Huh7.5 cells stably reconstituted for TLR3 expression (Huh7.5-TLR3) were transfected with an empty vector (“vector”) or a plasmid vector encoding WT or the indicated mutant human TRIM56, along with internal control plasmid pRL-TK and reporter plasmid IFN-β-Luc, followed by mock stimulation of stimulation by poly-I:C for 8 h and dual luciferase reporter assay. (*Lower* panel) Immunoblot analysis of the expression of the vectors encoding WT TRIM56-V5 and its Ser^469^Ala, Ser^471^Ala, or Ser^475^Ala single mutant, or Ser^469^A+Ser^471^A+Ser^475^A triple mutant, respectively (using mouse anti-V5 mAb) in HEK293 cells. Statistical analysis was performed between the indicated groups after poly-I:C stimulation. Single, double, and triple asterisks denote that statistical differences exist with a *p* value of <0.05, <0.01, and <0.001, respectively. N.S., not statistically significant; pIC, poly-I:C. IFN, interferon; TLR, Toll-like receptor; TRIM56, tripartite-motif protein-56.

In our efforts to understand the mechanisms by which the C-terminal portion of TRIM56 regulates TLR3 signaling, four serine residues conserved between human and murine TRIM56 (Fig. 5*A*) caught our attention. These are listed on PhosphoSitePlus as phosphorylation sites compiled from previous global-scale proteome profiling studies. Specifically, the cluster of Ser^469^, Ser^471^, and Ser^475^ (Ser^448^, Ser^450^, and Ser^454^ in mTRIM56) and Ser^710^ (Ser^689^ in mTRIM56) fall into aa 434-519 and aa 693-750, respectively, regions our earlier studies found to be critical for TRIM56 to potentiate TLR3 signaling (Figs. 3*A* and 4) (14). Of note, previous studies on post-translational modifications (PTMs) of TRIMs have been largely focused on ubiquitination due to the fact that TRIMs bear E3 ligase activity and they tend to catalyze the reaction on themselves, which is believed to be important for their functions (24, 25). Recently, phosphorylation-dependent regulation of TRIM protein activity is being recognized—an example is tyrosine phosphorylation of TRIM21, which was suggested to modulate TLR3/4-mediated IFN induction (26).

To determine the effect of putative Ser^710^ phosphorylation of TRIM56, vectors encoding phospho-deficient (Ser^710^Ala) and phospho-mimetic (Ser^710^Asp) mutants of hTRIM56-V5 were constructed in pcDNA3.1 backbone and were confirmed to express successfully in transiently transfected cells, comparable to V5-tagged, hTRIM56-WT (positive control) and the Δ693-750 mutant (negative control) (Fig. 5*B*). We found the Ser^710^Ala mutation significantly (*p* < 0.01) curtailed hTRIM56-mediated enhancement of poly-I:C–stimulated IFN-β promoter activity in HEK293-TLR3 cells, to similar extent as Δ693-750, suggesting Ser^710^ is important for TRIM56 to function as a positive regulator of TLR3 signaling (Fig. 5*C*, left). Likewise, an alanine substitution at Ser^689^ (Ser^689^Ala) in mTRIM56, the counterpart of Ser^710^ in hTRIM56, also blunted mTRIM56’s ability to augment the IFN-β promoter activation *via* the pathway (Fig. 5*C*, right).

To further investigate the effect of putative Ser^710^ phosphorylation on regulation of TLR3 signaling by TRIM56, we transiently expressed WT TRIM56 and Ser^710^ mutants, respectively, in HeLa-shT56-093 #9 cells that were stably transduced with an short hairpin (shRNA, shT56-093) specifically targeting the 3′UTR of hTRIM56 transcript. Because our WT- and mutant TRIM56-encoding constructs do not have the 3′UTR sequence inserted, they could be introduced back into TRIM56-depleted cells without interference by stably integrated shT56-093. As shown in Figure 5*D* (left), we confirmed by immunoblotting that HeLa-shT56-093 #9 was the best knockdown clone in which TRIM56 had been depleted the most (by ∼95%, lane 9) compared with parental HeLa (lane 1) or HeLa cells bearing a non-targeting, scrambled control shRNA (HeLa-shCtrl, lane 2). Expectedly, poly-I:C–induced IFN-β promoter activity was almost ablated in HeLa-shT56-093 #9 compared with HeLa-shCtrl cells (Fig. 5*D*, right, compare shCtrl and shT56-093 #9 cells, both transfected with empty vector). Mirroring the results obtained form HEK293-TLR3 cells (Fig. 5*C*), in HeLa-shT56-093 #9 cells, overexpression of WT TRIM56, but not Ser^710^Ala or Δ693-750, significantly augmented TLR3 signaling to activation of the IFN-β promoter, compared to cells transfected with empty vector (Fig. 5*D*, right). In addition, we found the phospho-mimetic Ser^710^Asp mutant acted as effectively as WT TRIM56, with both exhibiting statistically higher activity than the phospho-dead Ser^710^Ala mutant (Fig. 5*D*, right). Of note, these TRIM56 mutants exhibited the same functional pattern when tested in HeLa-shCtrl cells (Fig. S3). Taken together, these data illustrate that depletion of endogenous WT TRIM56 did not alter the effects of exogenously introduced TRIM56-Ser^710^Ala or TRIM56-Ser^710^Asp on TLR3 signaling. They collectively establish that putative phosphorylation at Ser^710^ is critical for TRIM56 to positively regulate TLR3 signaling.

Next, we investigated the potential involvement of phosphorylations on the Ser^469^, Ser^471^, and Ser^475^ clusters. Toward this end, alanine mutation at each serine alone or all three sites in combination were performed. As shown in Figure 5*E*, our results showed that TRIM56 bearing Ser^471^Ala or Ser^475^Ala single mutation, or Ser^469^Ala+Ser^471^Ala+Ser^475^Ala triple mutations, failed to enhance TLR3 signaling (*p* < 0.05, 0.001, and 0.01, respectively), while Ser^469^Ala was slightly impaired for such ability (not statistically significant if compared with WT TRIM56 though), indicating a critical role for putative phosphorylation of TRIM56 at Ser^471^ and Ser^475^ in promoting TLR3 signaling. Collectively, our data have identified three putative phosphorylation sites, Ser^710^, Ser^471^, and Ser^475^, with each playing an important part in TRIM56-mediated enhancement of TLR3 signaling.

### Alanine mutations on key putative phosphorylation sites or deletion of the Coiled-coil domain undermine the ability of TRIM56 to augment TLR3-dependent establishment of an antiviral state

**Figure 6:**
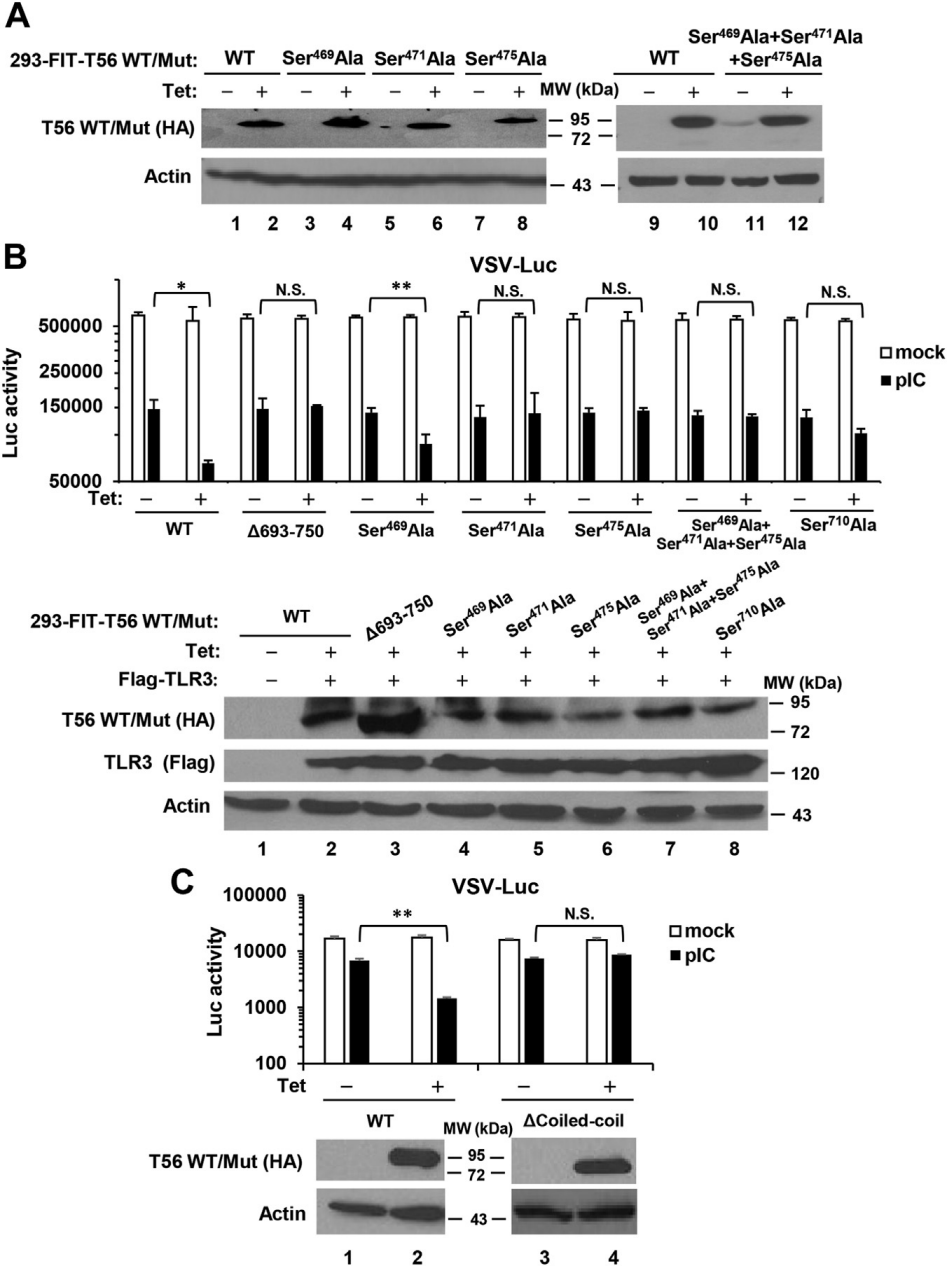
**Impact of alanine substitution of key putative phosphorylation sites or deletion of the Coiled-coil domain on TRIM56-mediated augmentation of TLR3-dependent establishment of an antiviral state.***A*, characterization of HEK293-FIT-derived cell lines that conditionally express indicated phospho-dead TRIM56 mutant in a Tet-inducible manner. Immunoblot analysis of the expression of WT HA-TRIM56 and various mutant TRIM56 (using mouse anti-HA mAb) in 293-FIT-T56 WT/Mut cells with (+Tet) or without (−Tet) Tet addition for 48 h. Actin serves as a loading control. *B*, (*Upper* panel) HEK293-FIT-derived cells conditionally expressing WT HA-TRIM56 and its mutants at the indicated putative phospho-sites with (+Tet) or without (−Tet) Tet, were reconstituted with TLR3 and mock-stimulated or stimulated by poly-I:C, followed by infection with VSV-Luc (MOI = 0.1). At 6 h postinfection, cells were lysed for firefly luciferase assay. (*Lower* panel) Immunoblot analysis of expression of the induced WT and mutant HA-TRIM56 (using mouse anti-HA mAb) and reconstituted Flag-tagged TLR3 (using mouse anti-flag mAb) in the 293-FIT-T56 WT/Mut cells before VSV-Luc infection. *C*, HEK293-FIT–derived cells conditionally expressing WT HA-TRIM56 or the Coiled-coil domain deletion mutant (ΔCoiled-coil) cultured with (+Tet) or without (−Tet) Tet were reconstituted with TLR3 and mock-stimulated or stimulated by poly-I:C, followed by infection with VSV-Luc (MOI = 0.1). At 4 h postinfection, cells were lysed for firefly luciferase assay. (*Lower* panel) Immunoblot analysis of the expression of WT HA-TRIM56 and the ΔCoiled-coil mutant (using mouse anti-HA mAb) in 293-FIT-T56 WT/ΔCoiled-coil cells before VSV-Luc infection. Statistical analysis was performed between poly-I:C-treated −Tet and +Tet cells. Single and double asterisks denote that statistical differences exist with a *p* value of <0.05 and <0.01, respectively. N.S., not statistically significant; pIC, poly-I:C. TLR, Toll-like receptor; TRIM56, tripartite-motif protein-56; VSV, vesicular stomatitis virus.

To further investigate the impact of the putative phosphorylation sites on TRIM56 regulation of TLR3-dependent antiviral responses, we created HEK293-FIT–derived cell lines with Tet-inducible expression of each HA-TRIM56 mutant. The mutants included Ser^469^Ala, Ser^471^Ala, Ser^475^Ala, Ser^710^Ala, and the Ser^469^Ala+Ser^471^Ala+Ser^475^Ala triple mutant—a total of 5. As shown in Figure 6*A*, there was negligible expression of mutant HA-TRIM56 when these cell lines were cultured in the absence of Tet (Fig. 6*A*, odd-numbered lanes, data for Ser^710^Ala not shown), while robust expression of each mutant HA-TRIM56, at levels comparable to each other and their WT version, was detected upon Tet addition (Fig. 6*A* and lower panel of 6B (including data on Ser^710^Ala (+Tet) cells).

Having characterized the HEK293-FIT–derived cell lines conditionally expressing WT or the five site-specific phospho-dead mutant forms of HA-TRIM56, each of these, along with that with conditional expression of the Δ693-750 mutant (17) (as a negative control which loses the ability to augment TLR3 response), was reconstituted with Flag-TLR3 by transient transfection and cultured with (+Tet) or without (−Tet) to induce or repress the expression of HA-tagged WT or mutant TRIM56. The cells were then stimulated by poly-I:C to induce antiviral state *via* the TLR3 pathway or mock-stimulated, followed by challenge by a recombinant vesicular stomatitis virus (VSV) expressing firefly luciferase (VSV-Luc). In mock-stimulated groups regardless of cell lines and Tet addition status, VSV-Luc replicated with similar efficiency as represented by the virus-encoded firefly luciferase activity (Fig. 6*B*, empty bars), which is in line with our previous report that TRIM56 overexpression itself had no demonstrable impact on VSV replication (15). Stimulation by poly-I:C induced an antiviral state in all seven cell lines without Tet addition (−Tet, *i.e.*, no induction of WT or mutant TRIM56) to similar extent among WT and the six mutant TRIM56 cell lines, leading to a ∼75% reduction in VSV-Luc replication compared with mock-stimulated cells (Fig. 6*B*, compare filled bars with empty bars, −Tet conditions). By contrast, in 293-FIT-T56 (+Tet) cells induced for WT HA-TRIM56 expression, significantly (*p* < 0.05) more inhibition by poly-I:C (by 88%) on VSV-Luc replication was observed, consistent with our previous report that TRIM56 enhances TLR3-dependent antiviral response (14). This phenomenon was also found in the TRIM56-Ser^469^Ala mutant expressing cells (+Tet) which exhibited 85% decrease in VSV-Luc replication by poly-I:C, suggesting that TRIM56-Ser^469^Ala retains the ability to promote TLR3-dependent antiviral response. On the contrary, cells expressing TRIM56-Δ693-750, -Ser^471^Ala, -Ser^475^Ala, -Ser^469^Ala+Ser^471^Ala+Ser^475^Ala, and -Ser^710^Ala all failed to enhance TLR3-dependent antiviral response, since there was no further significant decrease in VSV-Luc replication in poly-I:C-stimulated, +Tet cells compared with stimulated, -Tet cells for each of these mutants (Fig. 6*B*). Similar results were obtained when we created and examined 293-FIT-T56-ΔCoiled-coil cells with conditional expression of the Coiled-coil deletion mutant (Fig. 6*C*)—these cells cultured in the presence of Tet lost the ability to heighten poly-I:C-stimulated antiviral response against VSV-Luc, as opposed to 293-FIT-T56-WT cells with Tet in culture medium. In aggregate, data from these experiments directly link three putative phospho-acceptor sites Ser^471^, Ser^475^, and Ser^710^ in the C-terminal portion and the Coiled-coil domain to the ability of TRIM56 to promote TLR3-dependent antiviral responses.

### Biphasic phosphorylation at Ser^471^ and Ser^475^ residues of TRIM56 following engagement of the TLR3 pathway

We next sought to determine if indeed the key serine residues we identified critical for TRIM56’s function undergo phosphorylation upon evocation of TLR3 signaling. To this end, we first tried to detect phosphorylations at Ser^471^ and Ser^475^ by performing IP using anti-TRIM56 polyclonal antibody (pAb) followed by immunoblotting using an anti-phosphoserine antibody. Unfortunately, this approach turned out to be fruitless due to the well-known low specificity of anti-phosphoserine antibody. We then attempted to develop pAbs specifically reacting to phospho-Ser^471^, -Ser^475^, and -Ser^710^. Analysis of the TRIM56 protein sequence predicted that residues 371-484, which encompass Ser^471^ and Ser^475^, constitute a disordered region that is relatively hydrophilic, while its C-terminal tail region is much less so (Fig. S4). We encountered great difficulty in synthesizing a C-terminal tail peptide immunogen in which Ser^710^ falls—the yield was low, and its purity poor, most likely due to the overall hydrophobic nature of this portion of the protein. Thus, we focused our efforts on Ser^471^ and Ser^475^, for which we successfully developed phospho-specific antibodies.

**Figure 7:**
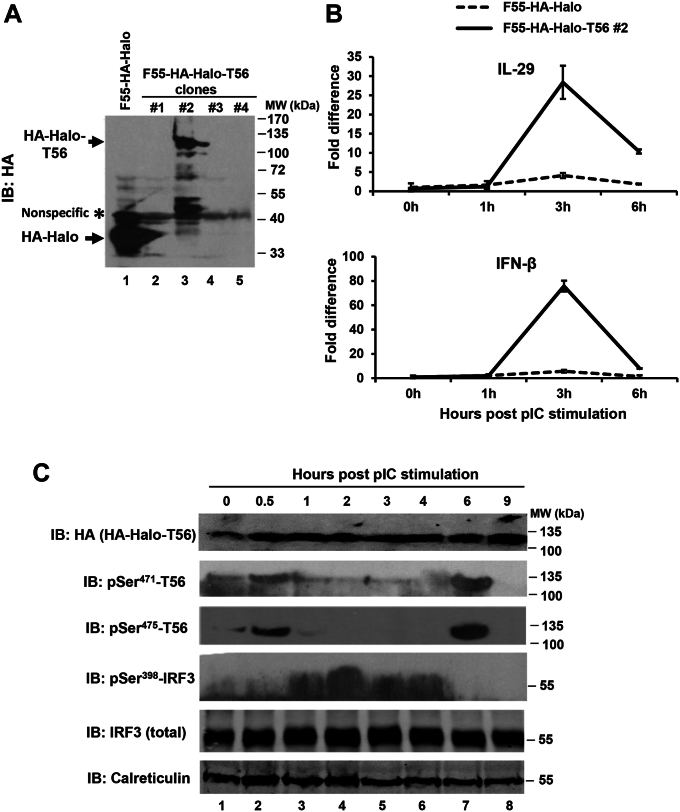
**Biphasic phosphorylation of TRIM56 at Ser**^**471**^**and Ser**^**475**^**and induction of IFNs following extracellular poly-I:C stimulation of HT1080-F55–derived cells expressing HA-Halo–tagged TRIM56.***A*, immunoblot analysis of the expression of HA-Halo–tagged TRIM56 or HA-Halo tags alone (using mouse anti-HA mAb) in HT1080-F55–derived cell clones stably transfected with HA-Halo-T56 (F55-HA-Halo-T56 clones) in comparison with cells expressing HA-Halo tags alone (F55-HA-Halo). Asterisk denotes a nonspecific band that served as a loading control. HA-Halo and HA-Halo tagged T56 are indicated using *arrows*. *B*, qRT-PCR analysis of the abundance of IL-29 (*upper* panel) or IFN-β (*lower* panel) transcript in F55-HA-Halo (*dashed* line) and F55-HA-Halo-T56 clone #2 (*solid* line) at indicated times post stimulation by 50 μg/ml poly-I:C. *C*, immunoblot analysis of the expression of the indicated proteins in F55-HA-Halo-T56 #2 cells at indicated time points post stimulation of 10 μg/ml poly-I:C. Cell lysates were immunoblotted (IB) with mouse anti-HA mAb (for total HA-Halo tagged T56), pAb specific for TRIM56 phosphorylated at Ser^471^ or Ser^475^, rabbit anti-pSer^398^-IRF3, and rabbit anti-IRF-3 pAb (for total IRF3). Calreticulin serves as a loading control. IFN, interferon; IRF, interferon regulatory factor; TRIM56, tripartite-motif protein-56.

Our initial trials with the p-Ser^471^ and p-Ser^475^ pAbs in immunoblotting suggested these could not readily detect phosphorylation of endogenous TRIM56, most likely because the abundance of the protein is low to moderate at best (14). Therefore, we examined whether ectopically expressed TRIM56 undergoes serine phosphorylations at residues 471 and 475 when TLR3 signaling is elicited. To achieve this, we created a human fibrosarcoma HT1080-derived cell line that stably expresses HA-Halo-tandem–tagged TRIM56 (F55-HA-Halo-T56 #2) and as a negative control, HT1080 cells stably expressing the HA-Halo tags only (F55-HA-Halo) (Fig. 7*A*). HT1080 cells were selected because they harbor an intact TLR3 signaling pathway that enables considerable IFN and ISG induction in response to extracellular dsRNA stimulation (27). Analysis of the kinetics of the IFN response post poly-I:C treatment, as revealed by qRT-PCR quantification of the transcript for IFN-β and IL-29, found expression of both types of IFNs peaked at ∼3 h, with F55-HA-Halo-T56 #2 cells exhibiting strikingly greater response than control F55-HA-Halo cells (Fig. 7*B*). These data show that TRIM56 boosts TLR3 signaling in HT1080 cells, as it does in HEK293-TLR3, HeLa, and Huh7.5-TLR3 cells (Fig. 5) (14) and support the use of F55-HA-Halo-T56 #2 cells for interrogating the status of TRIM56 phosphorylation in the context of TLR3 signaling.

Immunoblotting analyses demonstrated that while total HA-Halo-T56 levels remained steady, phosphorylations of the protein at Ser^471^ and Ser^475^ followed a biphasic pattern in response to poly-I:C stimulation and kinetics of the two phosphorylation events tracked together (Fig. 7*C*). p-Ser^471^ and p-Ser^475^ both were first detected at ∼0.5 h and waned quickly, returning to background levels by ∼1 h. A second phosphorylation event at both serine residues showed up at ∼6 h and did not hold long either—there was no detectable of p-Ser^471^ and p-Ser^475^ at 9 h. Interestingly, phosphorylation of IRF3 took place between the two spikes of TRIM56 phosphorylations—most detectable from ∼1 h to ∼4 h and cresting at ∼2 h. Notably, maximal IRF3 phosphorylation preceded the peak induction of IFN-β and IL-29 mRNAs (at ∼3 h, Fig. 7*B*), consistent with the established paradigm regarding transcriptional control of type I and III IFNs by IRF3. Taken together, these data provide direct evidence suggesting that transient phosphorylations of TRIM56 at Ser^471^ and Ser^475^ occur during very early and late phases of the TLR3 signaling cascade.

### TRIM56’s putative phosphorylation site Ser^710^ near the C-terminus, but not the Coiled-coil domain, is required for its association with TRIF

We next endeavored to characterize the mechanism(s) by which the (putative) phosphorylation and its Coiled-coil domain control the capacity of TRIM56 to promote TLR3 signaling. Given that TRIM56 can form a complex with TRIF (Fig. 2*B*) (14) and that Ser^710^ is located in the region (aa 621–750) critical for the TRIM56–TRIF interaction (14), we asked if a phospho-dead mutant, Ser^710^Ala, would modulate the ability of TRIM56 to associate with TRIF.

**Figure 8:**
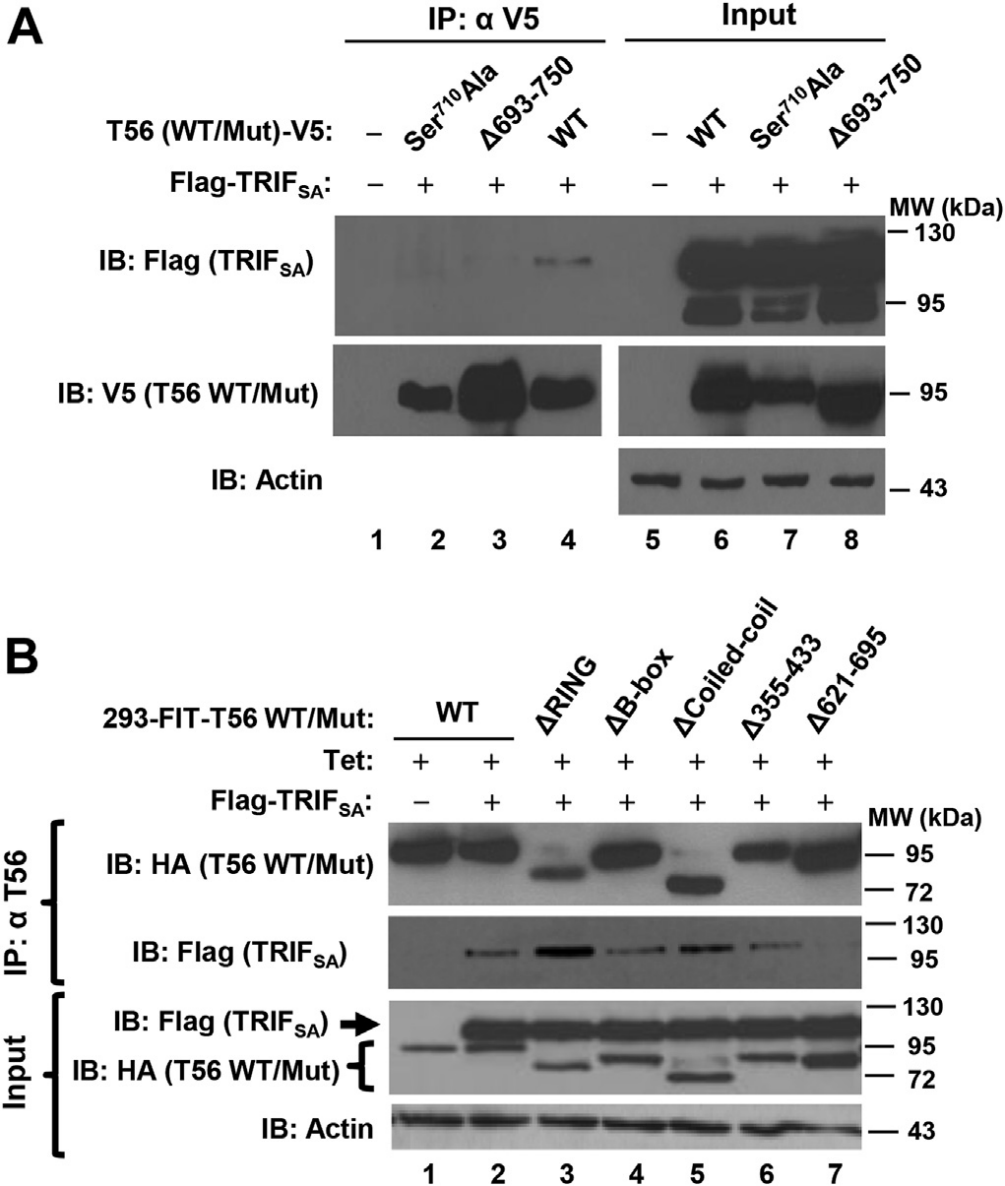
**Characterization of the interactions of various TRIM56 mutants with TRIF.***A*, co-immunoprecipitation (co-IP) analysis of the association of Flag-TRIF_SA_ and indicated TRIM56-V5 WT/mutant in cotransfected HEK293 cells. Cell lysates were immunoprecipitated (IP) with anti-V5, followed by immunoblotting (IB) with anti-Flag or anti-V5 (lanes 1–4). Immunoblotting of the expression of Flag-TRIF_SA_ and TRIM56-V5 (WT or indicated mutant) in cell lysates are shown in lanes 5-8. Note that TRIF_SA_ (TRIF lacking the C-terminal RHIM motif) was used to avoid the strong apoptotic effect of WT TRIF. *B*, Co-IP in HEK293-FIT-T56 WT/mut cells co-expressing Flag-TRIF_SA_ and WT or indicated mutant HA-TRIM56 (induced by addition of Tet (+Tet)). Cell lysates were IP with anti-TRIM56, followed by IB with anti-Flag or anti-HA. The *bottom* blot shows expression of HA-TRIM56 or the indicated mutant and Flag-TRIF_SA_ in cell lysates. Actin serves as loading controls. TRIM56, tripartite-motif protein-56.

Co-IP experiments showed that the Ser^710^Ala mutant, like Δ693-750, did not interact with TRIF (Fig. 8*A*). Because the Ser^710^Ala mutant lost the ability to enhance poly-I:C–induced IFN response (Fig. 5) and cellular antiviral state (Fig. 6*B*), we conjecture that putative phosphorylation at Ser^710^ facilitates the TRIM56–TRIF interaction, thereby potentiating TLR3-dependent antiviral response. Next, we asked if the Coiled-coil domain, which was found important for positively regulating TLR3-dependent antiviral responses (Figs. 3, 4, and 6*C*), is also indispensable for the association of TRIM56 with TRIF. As shown in the co-IP experiments with 293-FIT-T56 WT/Mut cells (Fig. 8*B*), the ΔCoiled-coil mutant could still form a complex with TRIF (contrary to the Δ621–695 mutant), indicating that the Coiled-coil domain is not required. Interestingly, under the same experimental conditions, the Δ355-433 mutant retained the ability to associate with TRIF (Fig. 8*B*), in line with its activity in promoting TLR3 signaling (Fig. 3) and excluding that this region of TRIM56 mediates TRIF binding. Remarkably, the ΔRING mutant exhibited stronger binding to TRIF than WT TRIM56, which could explain why Tet-regulated expression of TRIM56-ΔRING promoted greater TLR3 response than that of WT TRIM56 in this cell type and expression system (HEK293-FIT) (Fig. 3*B*).

## Discussion

In this study, we have conducted a comprehensive mutational analysis of TRIM56 to get a fuller picture of the molecular determinants that govern its positive regulation of the TLR3 pathway. Previously, we have reported that the integrity of TRIM56’s C-terminal tail portion (encompassing ∼130 residues) is critical; deletion of residues 621-695 or 693-750 abrogated the augmentation of TLR3 signaling and correlated with a loss in the interaction of TRIM56 with TRIF, the sole TLR3 adaptor. In contrast, TRIM56 mutants devoid of the E3 ligase activity or lacking the entire RING domain remained functional, indicative of a noncanonical mechanism (14). Extending these previous findings, the current study has identified three additional regions in human TRIM56 as indispensable, including the entire Coiled-coil domain and two fragments spanning residues ∼434-519 and 515-610, respectively. The latter results hint that an extended C-terminal portion—approximately two fifths of TRIM56—is required for this protein to function as a positive regulator of TLR3 signaling. Interestingly, bulk of this extended region is occupied by the NHL (NCL-1, HT2A, and LIN-41)-like repeats of TRIM56 (consisting of residues 492-753, Fig. S1) (18), which, according to published data on NHL of other proteins, adopt a six-bladed β-propeller structure known to be involved in protein–RNA and/or protein–protein interactions (18, 28). Indeed, parts of the NHL-like domain of TRIM56 are important for interacting with TRIF (see also below) (14) and flavivirus RNA (19). Since deletion of several different parts of the NHL-like domain invariably nullified the ability of TRIM56 to heighten TLR3 responses, we posit that the structural integrity of the NHL-like, rather than its primary sequence, is critical. This can also be said regarding the requirement for the Coiled-coil domain, as evidenced by the same deleterious effect observed with two TRIM56 mutants bearing deletion in non-overlapping regions of the Coiled-coil domain.

Almost immediately upstream of the NHL-like and within this extended C-terminal portion of TRIM56 essential for augmenting TLR3 responses lie a clusters of conserved serine residues, Ser^469^, Ser^471^, and Ser^475^, that are putative phosphorylation sites. Notably, these three are within the boundaries of an intrinsically disordered region (aa 371–484) predicted to be highly hydrophilic (Fig. S4), features conducive for PTMs (29). By alanine substitution at each site resembling phospho-dead mutation, we show Ser^471^ and Ser^475^ are both essential for TRIM56 to enhance TLR3-dependent IFN-β promoter activation and induction of a cellular antiviral state, while Ser^469^ is not. Importantly, by generating phospho-specific antibodies, we provide direct evidence of transient phosphorylations at Ser^471^ and Ser^475^ following TLR3 ligand stimulation. Interestingly, the kinetics of this PTM of TRIM56 at both residues tracked together, indicating a coordinated regulation. Moreover, both exhibited a biphasic pattern, occurring at very early (∼0.5–1 h) and late (∼6 h) stages of TLR3 signaling cascade. Currently, we do not know exactly why these phosphorylation events are fleet and highly dynamic. It is worth noting that the early phase of phosphorylations at Ser^471^ and Ser^475^ preceded that of IRF3 and coincided, if not earlier, with the TRIM56–TRIF interaction, suggesting they are at a proximal (early) step of TLR3 signaling. Future studies are warranted to investigate which kinase(s) is responsible for phosphorylating TRIM56 at these serine residues, precisely how these early Ser^471^ and Ser^475^ PTM of TRIM56 modulate TLR3 signaling and what the biological significance is as to the late phase TRIM56 phosphorylations.

Along with the PTM line, our study additionally suggests putative phosphorylation at a conserved serine residue in the C-terminal tail portion of TRIM56, Ser^710^, plays a vital role in boosting TLR3-mediated antiviral responses. This is supported by data showing that a phospho-dead mutant, Ser^710^Ala, was severely compromised for its activity. While we were unable to provide direct evidence of phosphorylation at this site due to technical challenges in producing a phospho-Ser^710^–specific antibody, our data did reveal that a phospho-mimetic mutant, Ser^710^Asp, maintained its function. Additional support to this is lent by experiments with murine TRIM56, which we show in this study also acts as an amplifier of TLR3-dependent IFN response. Importantly, we demonstrated that murine TRIM56 also depended on putative phosphorylation at Ser^689^, the mouse counterpart of Ser^710^ in human TRIM56, for this capacity, as Ser^689^Ala substitution significantly curtailed its activity. Since the TRIM56-Ser^710^Ala mutant lost its ability to associate with TRIF, as with Δ693-750 and Δ621-695, we favor the hypothesis that phosphorylation at this serine residue may orchestrate the overall folding or charge of the C-terminal tail to facilitate TRIM56’s interaction with TRIF (and possibly other signaling molecules), thereby supporting the TLR3 pathway-augmenting activity.

On the other hand, the current study reveals that the B-box and a central portion spanning residues 355-433 of TRIM56 are dispensable for its potentiation of TLR3 signaling. It is worth noting that the latter portion is the least conserved between human and murine TRIM56 and that compared to its human counterpart, this part of murine TRIM56 has two clusters of deletions totaling 21 aa that account for the bulk length difference (22-aa) between the two species (Fig. S1). Our data-uncovering murine TRIM56 also functions in heightening the TLR3 pathway which not only suggest this is an evolutionally conserved attribute but also help explain why deletion of aa 355-433 in human TRIM56 does not affect it activity of upregulating TLR3-dependent innate immunity.

Previously, we have proposed that the ability of TRIM56 to form a complex with the adaptor TRIF underlies its positive regulation of the TLR3 innate immune pathway (14). Extending our previous observation made in co-overexpression settings, we show here the two endogenous proteins associated with each other early after TLR3 engagement, ascertaining the physiologic relevance of our finding. Additional evidence revealed in the current study reinforces the notation that acting through TRIF is one main mechanism by which TRIM56 exerts its effects on the TLR3 pathway. Except for the ΔCoiled-coil mutant (see discussion below), TRIM56 mutants incapable of boosting TLR3 signaling (Ser^710^Ala, Δ621–695, and Δ693–750 (14)) all lost the capacity to physically associate with TRIF, while those with intact boosting function (ΔRING (14), ΔB-box, and Δ355–433) could still co-precipitate with the TLR3 adaptor. Also, in keeping with this proposed mechanism, we show here that enforced expression of TRIM56 also heightened NF-κB activation by extracellular dsRNA stimulation *via* TLR3 (which is TRIF-dependent) but not by IL-1β or TNF-α (neither of which depends on TRIF). Furthermore, our data revealed the same requirements of TRIM56 determinants for augmenting TLR3 signaling to both IRF3 and NF-κB branches, consistent with the consensus that TLR3 signaling bifurcates at TRIF.

A different mechanism likely underpins the contribution of the Coiled-coil domain, which we found to be required for TRIM56’s augmentation of TLR3 signaling. Coiled-coil domain of other TRIMs has been reported to mediate dimerization of TRIM molecules (23). Since we observed that the ΔCoiled-coil mutant of TRIM56 retained its ability to associate with TRIF despite having lost its activity of enhancing TLR3-dependent antiviral responses, we postulate that TRIM56 dimerization/multimerization is not a prerequisite for the TRIM56–TRIF interaction. Rather, the Coiled-coil domain may facilitate TRIM56 to serve as scaffolds to recruit other key signaling components to TRIF, thereby accelerating signal transduction downstream of TLR3. In future studies, it will be interesting to determine exactly how TRIM56’s scaffolding function conferred by its Coiled-coil domain contributes to positive regulation of TLR3 signaling.

In light of our previous findings that TRIM56 executes direct antiviral activities against specific positive- and negative-strand RNA viruses *via* overlapping and distinct molecular determinants (15, 17, 18, 19), the domain mapping data from our current study will aid a clearer understanding of the underlying biology of TRIM56’s direct antiviral and immunoregulatory properties. Importantly, our data suggest that these two activities of TRIM56 can be separated and could inform the development of novel antiviral therapies for infectious diseases. To name a few examples, the E3 ligase activity and RING domain, while critical for antiviral against flaviviruses and hCoV-OC43, are dispensable for restraining influenza viruses or amplifying TLR3-TRIF signaling. The central portion (aa 355–433), whereas required for the anti-flaviviruses activity, can be deleted without losing inhibitory effects on hCoV-OC43 and influenza viruses or compromising the positive regulation of TLR3-dependent innate immunity. The integrity of the C-terminal tail portion is essential for suppressing flaviviruses and augmenting TLR3 signaling but not required for the anti-hCoV-OC43 activity. As another example, a short C-terminal peptide derived from TRIM56 retains the anti-influenza activity (18) is predicted to avoid the protein’s unfavorable proinflammatory property based on data from the current study.

In summary, this study reveals that boosting TLR3-dependent innate immune responses is a conserved attribute shared by human and murine TRIM56 and extends the protein’s regulatory effects to TRIF-dependent activation of both IRF3 and NF-κB branches. In addition, this work demonstrates that an extended C-terminal portion and the Coiled-coil domain are both essential for the augmentation of TLR3 signaling by TRIM56. Further, we have discovered phosphorylations at Ser^471^, Ser^475^, and Ser^710^ (putative) all play crucial parts in this immunoregulatory activity. Mechanistically, we show that some but not all, of the TRIM56 determinants uncovered in this study operate *via* the interaction with TRIF. Together, our data provide novel insights into the mechanism of TRIM56-mediated positive regulation of TLR3-dependent innate antiviral immunity and highlight key roles for TRIM56 scaffolding and PTM *via* phosphorylation.

## Experimental procedures

### Plasmids

Plasmids were constructed by conventional PCR cloning techniques, as described elsewhere (17, 30, 31). To construct a murine TRIM56 (mTRIM56 or mT56) expression vector, the cDNA encoding mTRIM56 was reversely transcribed from total RNA of mouse hepatoma hepa1-6 cells and amplified by PCR using the following oligonucleotide primers, 5′-GAAGATCTATGAACTCCAAAGACTCCTCCCCAAC-3′ (forward) and 5′-GAATTCGCTGTCAGGAAACCTGACCCTAAAGA-3′ (reverse), followed by ligation into pEF6/V5-His-TOPO (Invitrogen). The resultant plasmid was designated pEF6-mTRIM56-V5His, in which mTRIM56 would be expressed in frame with C-terminal V5 and His6 tags. The plasmid encoding N-terminally 2 × HA-tagged human TRIM56 (here referred to as hTRIM56, hT56, TRIM56, or T56) in the pcDNA5/FRT/TO backbone (Invitrogen), pcDNA5/FRT/TO-HA-TRIM56, had been described previously (14). The pcDNA3.1-based plasmid vectors encoding C-terminally V5-His6-tagged WT TRIM56 (designated pcDNA3.1-TRIM56-V5His) and its E3 ligase-null mutant (Mut) CC21/24AA and various deletion mutants were described elsewhere (15). The Ser^469^Ala, Ser^471^Ala, Ser^475^Ala, Ser^710^Ala, or Ser^710^Asp individual mutations or Ser^469^Ala+Ser^471^Ala+Ser^475^Ala triple mutations were introduced into pcDNA3.1-TRIM56-V5His vector by inverse PCR mutagenesis, as was the Ser^689^Ala mutation into pEF6-mTRIM56-V5His. The primers used for PCR mutagenesis are listed in Table 1. The gene fragments coding for various mutant TRIM56 (Ser^469^Ala, Ser^471^Ala, Ser^475^Ala, Ser^710^Ala, Ser^710^Asp, or Ser^469^Ala+Ser^471^Ala+Ser^475^Ala) in pcDNA3.1 backbone were then transferred to pcDNA5/FRT/TO-HA backbone for subsequent use in establishing Tet-inducible cell lines (see below). For expression of HA- and HaloTag-tandem–tagged TRIM56 (referred to as HA-Halo-T56), the Met-2xHA tag sequence N-terminal to human TRIM56 coding sequence in the pcDNA5/FRT/TO-HA-TRIM56 construct was substituted with sequence that encodes Met-HA-Halo tags (without termination codon) and amplified by PCR from the N-terminal HaloTag vector (Promega) template, to result in the vector pcDNA5/FRT/TO-HA-Halo-TRIM56. As a control, a plasmid vector expressing HA-Halo-poly linker only was constructed by inserting cDNA sequence encoding a Met-HA-Halo tags fusion protein into the pcDNA5/FRT/TO backbone.

**Table 1:** Primers for PCR mutagenesis of human (hT56) and murine TRIM56 (mT56)

Mutation(s)	Primer sequences (5′->3′)
hT56-Ser^469^Ala	Forward: ATATCCCGGGAGCCCAGCCCAG Reverse: CGCCTTGAGCCTGCCTTTGAAC
hT56-Ser^471^Ala	Forward: GCTCGAGAGCCCAGCCCAGCCCT Reverse: AATTGACTTGAGCCTGCCTTTGA
hT56-Ser^475^Ala	Forward: GCTCCAGCGCTGGGGCCGAATCT Reverse: GGGCTCCCGGGAAATTGACTTGA
hT56-Ser^710^Ala	Forward: GCTCTCCTTGGAGACTTCCTG Reverse: TCCCTTCGGGTCCAGGATCAC
hT56-Ser^710^Asp	Forward: GATCTCCTTGGAGACTTCCTG Reverse: TCCCTTCGGGTCCAGGATCAC
hT56-Ser^469^Ala+Ser^471^Ala+Ser^475^Ala	Forward: GAGCCCGCTCCAGCCCTGGGGCCGAAT Reverse: TCGAGCAATAGCCTTGAGCCTGCCTTTGA
mT56-Ser^689^Ala	Forward: GCTCTTCTTGGTGACTTCCTAAC Reverse: TCCCTTGGGATCCAGTATCAC

pFlag-TRIF-mRHIM encoding a Flag-tagged, constitutively active form of human TRIF that unlike WT TRIF, no longer triggers apoptosis when ectopically expressed (here referred to as Flag-TRIF_SA_) (14, 32), pCMV1-Flag-TLR3 encoding Flag-tagged human TLR3 (33), and the various promoter reporter plasmids, pIFN-β-Luc (34), pIL-29-Luc (35), and PRDII-Luc (36) have been described. pRL-TK (Promega) was used as an internal control plasmid for normalization of transfection efficiency in reporter gene assay experiments. The identities of all plasmids were confirmed by DNA sequencing and bioinformatic analysis, as described elsewhere (37, 38, 39, 40).

### Cells

Cells were propagated essentially as described previously (18, 41). HeLa, Vero, HEK293, HEK293-TLR3 cells (HEK293 cells constitutively expressing Flag-tagged human TLR3) were maintained in Dulbecco’s Modified Eagle Medium supplemented with 10% fetal bovine serum, 100 U/ml of penicillin, and 100 μg/ml streptomycin. Human hepatoma Huh7.5 cells with stably reconstituted expression of Flag-tagged human TLR3 (Huh7.5-TLR3) were described previously (42). HEK293-T3Y-FH-T56 cells constitutively expressing Flag- and HA-tandem tagged human TRIM56 (FH-TRIM56) have been described (19). These were derived from HEK293 cells stably expressing low levels of TLR3-YFP (293-T3Y), by stable transduction of a replication-incompetent retroviral vector carrying FH-T56 and selection of puromycin-resistant cell pools. HEK293-Flp-In T-REx (FIT) cells with tetracycline (Tet)-inducible expression of HA-tagged, WT, and various mutant versions of TRIM56 were cultured as described previously (17). Herein, we created HEK293-FIT-derived cell lines with Tet-inducible expression of various phospho-dead TRIM56 mutants (Ser^469^Ala, Ser^471^Ala, Ser^475^Ala, Ser^710^Ala, or Ser^469^Ala+Ser^471^Ala+Ser^475^Ala) using the FIT expression system (Invitrogen) as described (14, 17). In brief, 293-FIT cells (Invitrogen) were co-transfected with the pOG44 plasmid (Invitrogen) encoding the Flp recombinase and the indicated TRIM56 mutant vector of pcDNA5/FRT/TO-HA backbone at 9:1 ratio, followed by stable selection of cells in hygromycin-containing medium (200 μg/ml). To induce the WT or mutant HA-TRIM56 expression in 293-FIT-derived cells (designated 293-FIT-T56 WT/Mut cells), cells were cultured in Tet-containing medium for 24 to 48 h. To establish cells with stable, constitutive expression of HA-Halo-T56 fusion protein or the HA-Halo control, respectively, we took advantage of HT1080-F55 cells (43), a human fibrosarcoma HT1080-derived clonal cell line that contains a single, integrated copy of the Flp-recombination target site. HT1080-F55 cells were co-transfected at 9:1 ratio, with pOG44 (Invitrogen) and pcDNA5/FRT/TO-HA-Halo-TRIM56 or pcDNA5/FRT/TO-HA-Halo, respectively, followed by selection with 200 μg/ml of Hygromycin. A clonal stable cell line, designated F55-HA-Halo-T56 #2, was found to express HA-Halo-T56 by immunoblotting with anti-HA tag antibody as well as anti-TRIM56 mAb and expanded for further analysis. Hygromycin-resistant colonies stably expressing HA-Halo tags were pooled, designated F55-HA-Halo, and used as a negative control for comparison with F55-HA-Halo-T56 #2.

### VSV, TLR ligands, and cytokines

A recombinant VSV expressing firefly luciferase, VSV-Luc, was generously provided by Sean Whelan (Harvard University). VSV-Luc stocks were prepared and titrated by a standard plaque assay, in Vero cells. Poly-I:C (or abbreviated as pIC) was purchased from Sigma. For stimulation of cells to activate the TLR3 pathway, poly-I:C was added directly into culture medium to mimic extracellular dsRNA at the concentration specified (ranging from 10 to 50 μg/ml). To stimulate cells with TNF-α and IL-1β (both from PeproTech), a final concentration of 10 ng/ml was administered to the culture medium for the indicated period.

### Antibodies against TRIM56

We previously have developed a rabbit pAb against TRIM56 (S537-2) by immunizing rabbits with a keyhole limpet hemocyanin–coupled peptide spanning residues 153-168 of human TRIM56 (15). To generate antibodies against the C-terminal region of TRIM56 that more sensitively detect the endogenous protein expressed at physiological levels, rabbits were immunized with a recombinant TRIM56 fragment encompassing the C-terminal 392 aa of human TRIM56 fused to maltose-binding protein (MBP-T56-C392) (19) *via* a fee-for-service at Proteintech. The resultant pAb was designated rabbit anti-TRIM56 S4091.

To develop monoclonal antibodies (mAbs) against TRIM56, BALB/c mice were given primary immunizations with 100 μg recombinant MBP-T56-C392 in complete Freund’s adjuvant (BD Difco) with secondary and tertiary boosting immunizations at 3-weeks intervals with 100 μg MBP-T56-C392 in incomplete Freund’s adjuvant (BD Difco) and PBS, respectively. This work had been approved by the Institutional Animal Care and Use Committee at the University of Tennessee Health Science Center. MAb-producing hybridomas were generated from immune BALB/c mouse spleen cells by standard PEG (PEG, MW 1500 Da) fusion (44) with cells from the P3x63-Ag8.653 (Ag8) plasmacytoma line (45). Fusions were performed 3 days after the last boost. TRIM56 IgG antibody-producing hybrids were identified by ELISA on MBP-T56-C392-coated microELISA plates (Dynes Technologies) after HAT selection (44) and immunoblotting of TRIM56-overexpressing HEK293 cell lysates. ELISA using MBP-coated plates was performed in parallel to exclude hybridoma lines producing MBP antibody. Two hybridoma lines specifically producing TRIM56 IgG antibody, 3A6 and 4C5, were subject to two rounds of limiting dilution cloning and kept. A third line (5C5) also secreted high levels of anti-TRIM56 mAb into culture supernatant, which was used for immunoblotting in some experiments. Unfortunately, we were unsuccessful in cloning this hybridoma line.

To generate pAbs specific for TRIM56 phosphorylated at Ser^471^ and Ser^475^, phosphopeptides corresponding to residues 467-480 (LKSI{pSer}REPSPALGPC) and 472-484 (REP{pSer}PALGPNLDGC) of human TRIM56, respectively, were synthetized, coupled to keyhole limpet hemocyanin and used to immunize rabbits (GenScript). Phospho-specific TRIM56 antibodies were separated from nonphospho antibodies in the hyperimmune sera by affinity purification.

### Reporter gene assay

IFN-β, IL-29, and PRDII promoter activities in transfected cells prior to and after indicated treatments were measured using dual-luciferase reporter assay as described (46, 47, 48, 49).

### RNA interference

To stably deplete endogenous TRIM56 without interfering with the introduction of exogenous protein by transfection of a TRIM56-expressing vector (containing the coding sequence of TRIM56 only), we employed an shRNA in the pLKO.1-puro backbone that specifically targets the 3′ UTR of hTRIM56 mRNA (Openbiosystems, referred to as pLKO.1-shT56-093). The target sequence is as follows: 5′-CGTCTTCTAGTGTGTGAGAAT-3′. Packaging of replication-incompetent lentiviral particles carrying the shRNA, infection, and selection of HeLa cells with puromycin were performed as described previously (17). Individual cell colonies having survived the selection were picked, expanded for further analyses. In parallel, puromycin-resistant pool of HeLa cells stably transduced with a nontargeting scrambled shRNA (HeLa-shCtrl) (17) was used as a negative control for comparison.

For transient knockdown of endogenous TRIM56 in 293-FIT-T56-WT/Mut cells without affecting the expression of exogenously, Tet-induced WT or mutant HA-TRIM56, an siRNA (siT56-UTR) specifically targeting the 3′ UTR of human TRIM56 transcript was employed, as described previously (17).

### RNA analyses

Extraction of total cellular RNA by TRIzol, cDNA synthesis by reverse transcription, and qPCR were implemented as described elsewhere (15, 48). The primers amplifying TRIM56-coding region and 3′UTR, respectively, were described previously (17). The primers amplifying IL-29 and IFN-β transcript, respectively, were described previously (50). The relative abundance of each target was normalized to that of 28S rRNA.

### Immunoprecipitation and immunoblotting

Cell lysates were prepared and subject to immunoprecipitation or/and immunoblot analysis as previously described (15, 46). The following pAbs and mAbs were utilized: rabbit anti-TRIM56 S4091 pAb (this study); rabbit anti-pSer^471^-TRIM56 and anti-pSer^475^ pAbs (this study); rabbit anti-ISG56 pAb (42); rabbit anti-TRIF pAb (51); rabbit anti-IRF-3 pAb (52); rabbit anti-pSer^398^-IRF3 (Upstate), rabbit anti-MBP pAb (New England Biolabs), mouse anti-TRIM56 mAbs (this study), mouse anti-HA tag mAb (Invivogen); mouse anti-V5 tag mAb (Invitrogen); mouse anti-Flag M2 mAb (Sigma); mouse anti-actin mAb and rabbit anti-calreticulin pAb (both form Sigma); peroxidase-conjugated secondary goat anti-rabbit and goat anti-mouse pAbs (Southern Biotech).

### Statistical analysis

Statistical analysis was carried out using students’ *t* test with the SPSS 11.5 software (https://www.ibm.com/products/spss-statistics) where appropriate, as described elsewhere (53, 54, 55)). All *p* values were two-tailed and *p* < 0.05 was regarded to be statistically significant. Error bars represent SDs.

## Data availability

All the data are contained within the article and supporting information. Data can be made available upon request of the lead contact.

## Supporting information

This article contains supporting information − Figures S1–S4 (56).

## Conflict of interest

The authors declare that they have no conflicts of interest with the contents of this article.
