# Supplementary material for: Key roles for phosphorylation and the Coiled-coil domain in TRIM56-mediated positive regulation of TLR3-TRIF–dependent innate immunity
Source: J Biol Chem. 2024 Mar 29;300(5):107249. doi: 10.1016/j.jbc.2024.107249 (PMC11067339; doi:10.1016/j.jbc.2024.107249)
Supplement: Supporting Figures S1–S4 [file mmc1.pdf]

Supplemental Figures

Key Roles for Phosphorylation and the Coiled-coil Domain in TRIM56-mediated Positive Regulation of TLR3-TRIF-dependent Innate Immunity

Benjamin M. Liu, Nan L. Li, Ruixue Wang, Xiaofan Li, Z. Alex Li, Tony N. Marion, and Kui Li

Fig. S1. Sequence alignment of murine and human TRIM56 proteins. The predicted RING, B-Box, Coiled-coil, and NHL-like domains are shaded yellow, green, blue, and grey, respectively. Residues that are missing in murine or human TRIM56 are shown in red letters.

Fig. S2. Transient expression of plasmid vectors encoding TRIM56 and its mutants. (A) Schematic representation of wild-type (WT) TRIM56 and its various deletion mutants. R, B and CC denote RING, B-box, and Coiled-coil domains, respectively. The C-terminal putative NHL-like repeats are depicted as filled blue boxes. **(B)** Immunoblot analysis of the expression of plasmid vectors encoding C-terminally V5-tagged, WT TRIM56 and the indicated mutants, respectively, in transiently transfected HEK293-TLR3 cells using mouse anti-V5 mAb. Actin served as a loading control.

Fig. S3. Impact of mutations of putative phosphorylation site Ser^{710} in the C-terminal portion of TRIM56 on activation of the IFN-β promoter downstream of TLR3 signaling.

HeLa cells stably expressing a non-targeting, scrambled control shRNA (HeLa-shCtrl) were transfected with an empty vector ("vector”) or a plasmid vector encoding WT or the indicated mutant human TRIM56, along with internal control plasmid pRL-TK and reporter plasmid pIFN-β-Luc, followed by mock stimulation or stimulation by poly-I:C for 8 h. Dual luciferase reporter assay was then performed. Statistical analysis was performed between the indicated groups after poly-I:C stimulation. Single and double asterisks denote that statistical differences exist with a *P* value of < 0.05 and < 0.01, respectively. N.S., not statistically significant. pIC, poly-I:C.

Fig. S4. Hydrophobicity scales assigned for different regions of human TRIM56 protein by ProtScale using the method described by Kyte & Doolittle (56). X-axis denotes aa position, while Y-axis values are scores that determine the relative hydrophobicity of aa residues. Note that residues 371-484 are predicted to be a disordered region.

Figure S1

Identities = 610/756 (81%), Positives = 659/756 (87%), Gaps = 23/756 (3%)

```
mouse   MNSKDSSPTLLEALSSDFLACKICLEQLHTPKTLPCLHTYCQDCLAQLDIGGQVRCPECR 60
human   MVSHGSSPSLLEALSSDFLACKICLEQLRAPKTLPCLHTYCQDCLAQLADGGRVRCPECR 60
        * *:.***:*******************::******************  **:*******

mouse   EIVPVPAEGVAAFKTNFFVNGLLDLVKARAPGDVHSGKPTCALCPLVGGKSSGGPATARC 120
human   ETVPVPPEGVASFKTNFFVNGLLDLVKARACGDLRAGKPACALCPLVGGTSTGGPATARC 120
        * ****.****:****************** **:::***:*********.*:********

mouse   LDCADDLCQACADGHRCSRQTHKHRVVDLVGYRAGWYDEEARERQASQCPQHPGEALCFL 180
human   LDCADDLCQACADGHRCTRQTHTHRVVDLVGYRAGWYDEEARERQAAQCPQHPGEALRFL 180
        *****************:****.***********************:********** **

mouse   CQPCSQLLCKDCRLGPHIDHPCLPLAEAVRSRKPGLEELLAGVDSNLVELEATRVAEKEA 240
human   CQPCSQLLCRECRLDPHLDHPCLPLAEAVRARRPGLEGLLAGVDNNLVELEAARRVEKEA 240
        *********::***.**:************:*:**** ******.*******:* .****

mouse   LALLREQAASVGTQVEEAAERILKSLLAQKQEVLGQLRALVEAAEEATRERLTKIERQEQ 300
human   LARLREQAARVGTQVEEAAEGVLRALLAQKQEVLGQLRAHVEAAEEAARERLAELEGREQ 300
        ** ****** ********** :*::************** *******:****:::* :**

mouse   VAKAAAAFARRVLSLGLEAEILSLEGAITQRLRQLQDAPWTSGPTRCVLPQLELHPGLED 360
human   VARAAAAFARRVLSLGREAEILSLEGAIAQRLRQLQGCPWAPGPAPCLLPQLELHPGLLD 360
        **:************* ***********:*******..**:.**: *:********** *

mouse   KNCHLLRLIFEEPKQSPKDSGKGGAGTQGGDEAQGQGDDRTKIGKQGGAQP------LTP 414
human   KNCHLLRLSFEE-QQPQKDGGKDGAGTQGGEESQSRREDEPKTERQGGVQPQAGDGAQTP 419
        ******** *** :*. **.**.*******:*:*.: :*..*  :***.**       **

mouse   KE----------------GKDQNPQEDDGVFIERGNRPNKKKKCKGRGKSVSREPSPILR 458
human   KEEKAQTTREEGAQTLEEDRAQTPHEDGGPQPHRGGRPNKKKKFKGRLKSISREPSPALG 479
        **                .: *.*:**.*   .**.******* *** **:****** *

mouse   PNLEGSGLLPRPVFSWSFPTRMPGDKRSPRITGLCPYGPQEILVADEQNRVLKRFSLNGD 518
human   PNLDGSGLLPRPIFYCSFPTRMPGDKRSPRITGLCPFGPREILVADEQNRALKRFSLNGD 539
        ***:********:*  ********************:**:**********.*********

mouse   YKGTVQVPEGCSPCSVAALQNAVAFSANAKLYLVSPDGEIQWRRSLSLTQSSHAVAAMPC 578
human   YKGTVPVPEGCSPCSVAALQSAVAFSASARLYLINPNGEVQWRRALSLSQASHAVAALPS 599
        ***** **************.******.*:***:.*:**:****:***:*:******:*.

mouse   GDRVAVSVAGHVEVYKKDGSLATRFIPGGKASRGQRALVFLTTSPQGNFVGSDWQQNSVV 638
human   GDRVAVSVAGHVEVYNMEGSLATRFIPGGKASRGLRALVFLTTSPQGHFVGSDWQQNSVV 659
        ***************: :**************** ************:************

mouse   FCDGLGQVIWEYKGPGLHGCQPGSVSVDKKGYIFLTLREVNKVVILDPKGSLLGDFLTAY 698
human   ICDGLGQVVGEYKGPGLHGCQPGSVSVDKKGYIFLTLREVNKVVILDPKGSLLGDFLTAY 719
        :*******: **************************************************

mouse   HGLEKPRVTTMVDGKYLVVSLSNGTIHVFRVRFPDS 734
human   HGLEKPRVTTMVDGRYLVVSLSNGTIHIFRVRSPDS 755
        **************:************:**** ***
```

Figure S2

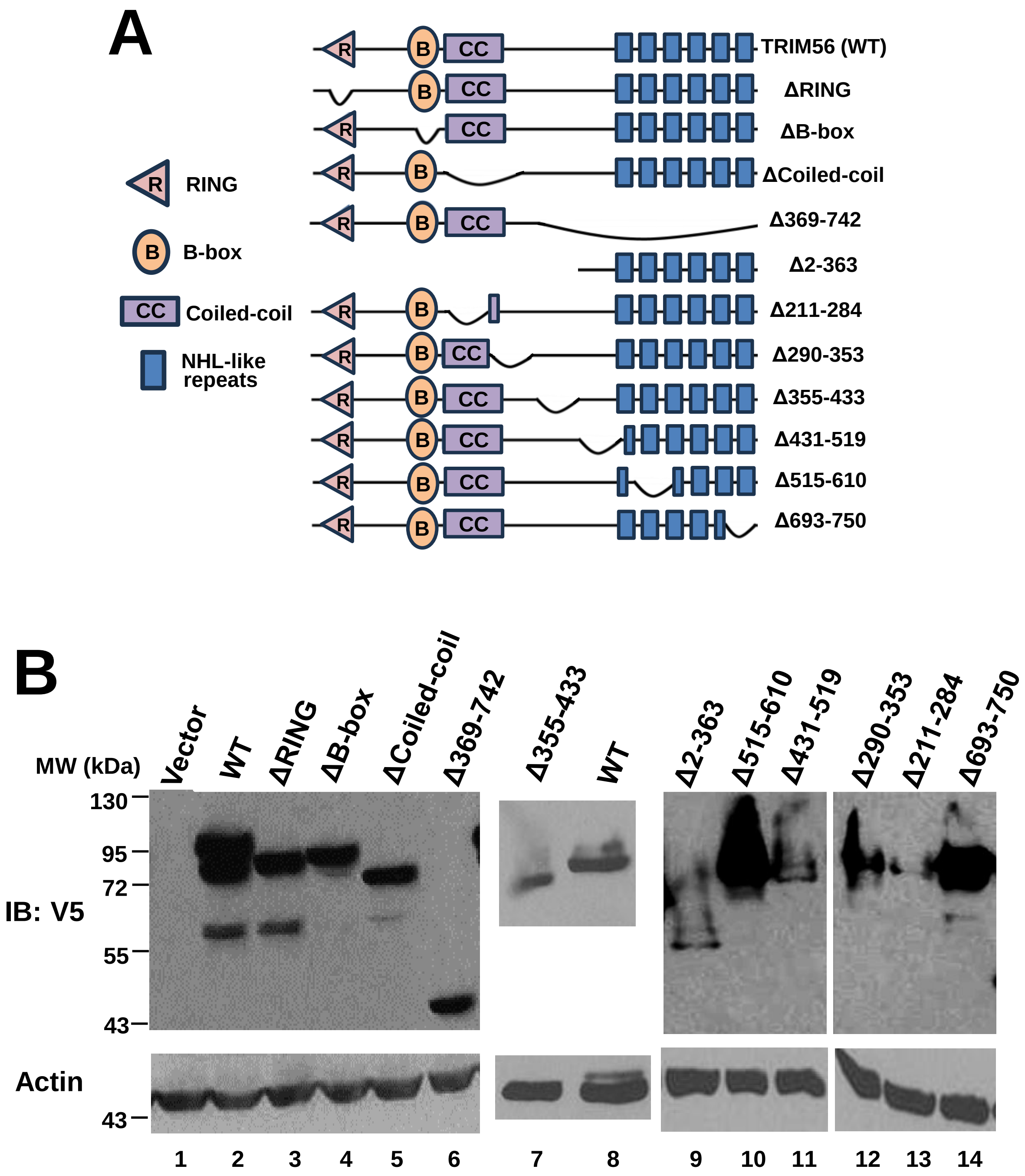
A
TRIM56 (WT)
ΔRING
ΔB-box
ΔCoiled-coil
Δ369-742
Δ2-363
Δ211-284
Δ290-353
Δ355-433
Δ431-519
Δ515-610
Δ693-750
RING
B-box
Coiled-coil
NHL-like repeats
B
MW (kDa)
Vector
WT
ΔRING
ΔB-box
ΔCoiled-coil
Δ369-742
Δ355-433
WT
Δ2-363
Δ515-610
Δ431-519
Δ290-353
Δ211-284
Δ693-750
130
95
72
55
43
IB: V5
Actin
43
1 2 3 4 5 6 7 8 9 10 11 12 13 14

Figure S3

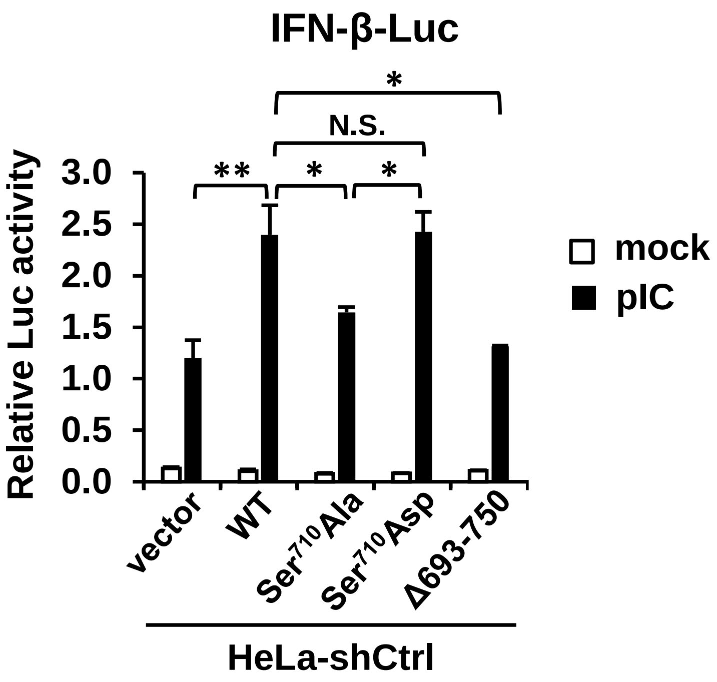
IFN-β-Luc
*
N.S.
**
*
*
Relative Luc activity
3.0
2.5
2.0
1.5
1.0
0.5
0.0
mock
pIC
vector
WT
Ser710Ala
Ser710Asp
Δ693-750
HeLa-shCtrl

Figure S4

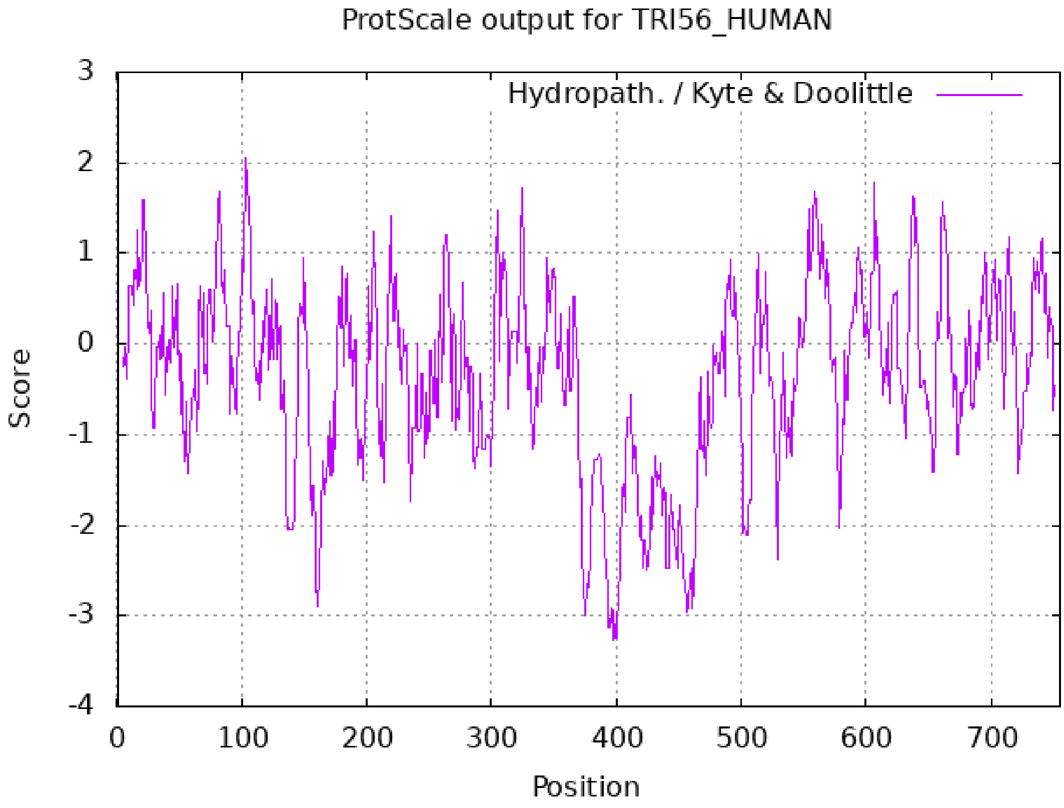
ProtScale output for TRI56_HUMAN
Hydropath. / Kyte & Doolittle
Score
3
2
1
0
-1
-2
-3
-4
0
100
200
300
400
500
600
700
Position
